# Improving the reliability of polygenic risk score-based prediction for cardiovascular and renal complications across ancestries in type 2 diabetes using Mondrian Cross-Conformal Prediction

**DOI:** 10.1371/journal.pcbi.1014670

**Published:** 2026-08-13

**Authors:** Edoh Kodji, Redha Attaoua, Mounsif Haloui, Camil Hishmih, Mirjam Seitz, Mark Woodward, Julie G. Hussin, Pavel Hamet, Johanne Tremblay

**Affiliations:** 1 Centre de Recherche, Centre Hospitalier de l’Université de Montréal (CRCHUM), Montréal, Québec, Canada; 2 Imperial College London, London, United Kingdom; 3 The George Institute for Global Health, London, United Kingdom; 4 The George Institute for Global Health, Sydney, Australia; 5 Research Center, Montreal Heart Institute, Montréal, Québec, Canada; University of Alabama at Birmingham, UNITED STATES OF AMERICA

## Abstract

Polygenic risk scores (PRS) developed in European populations often show reduced predictive performance in non-European populations, limiting their clinical utility. This lack of transferability across ancestries remains a major challenge in genomic medicine and raises concerns about health equity. We aimed to evaluate whether uncertainty-aware prediction, implemented through Mondrian Cross-Conformal Prediction, improves the performance and reliability of polygenic risk score-based predictions across ancestries for nephropathy, stroke, and myocardial infarction in individuals with type 2 diabetes in a multi-ethnic cohort. We leveraged Mondrian Cross-Conformal Prediction (MCCP), an uncertainty quantification framework, combined with logistic regression applied to a multi-polygenic risk score (multiPRS) to predict the risk of nephropathy, stroke, and myocardial infarction in individuals with type 2 diabetes. Two training frameworks were evaluated: one using 4,098 individuals with type 2 diabetes of European ancestry from the ADVANCE trial for training and 17,574 White British, 1,145 South Asian, and 749 African UK Biobank participants for testing; and another using the 17,574 White British UK Biobank participants for training and the South Asian and African participants for testing. Logistic regression provided robust baseline performance across populations. On top of this baseline, MCCP did not improve performance but added capabilities absent from probability-based stratification: for each individual, it issued a prediction together with an explicit confidence and credibility level; it allowed a tolerated error level to be set in advance and delivered prediction sets respecting it in the majority of settings; and it flagged individuals for whom no reliable prediction could be made. Applying MCCP to PRS-based prediction thus enables uncertainty-aware risk stratification and improves the reliability of risk prediction across ancestries, providing a more equitable framework for clinical use.

## Author summary

Type 2 diabetes is a major global health concern, often leading to serious complications such as heart disease, stroke, and kidney failure. Identifying individuals at high risk of developing these complications is essential for improving prevention and treatment strategies. Polygenic risk scores, which use genetic information to estimate disease risk, have shown promise but are typically developed using data from individuals of European ancestry. As a result, their performance is often reduced in other populations, raising concerns about their clinical applicability and fairness. In this study, we showed that incorporating a method that accounts for prediction uncertainty can improve the reliability of genetic risk prediction across diverse populations. Using data from large clinical and population-based cohorts, we show that this approach allows the acceptable error level to be set in advance and helps identify individuals for whom the model is less certain. This additional information may support more informed clinical decision-making. Our findings highlight a potential strategy to improve the equitable use of genetic risk prediction in multi-ethnic populations.

## Introduction

Diabetes burden is increasing worldwide, affecting individuals across all ages, continents, and communities. In 2024, 589 million adults were living with diabetes (11.1% prevalence), a number projected to reach 853 million by 2050 [1]. Type 2 diabetes (T2D) accounts for 90–95% of all diabetes cases [2] and is associated with severe complications, including cardiovascular disease (CVD) and renal failure, which substantially contribute to morbidity and healthcare burden. Early identification of individuals at high risk of developing such complications is essential to enable targeted prevention and therapeutic interventions [3,4].

In recent years, polygenic risk scores (PRS) have emerged as promising tools to predict susceptibility to complex diseases like T2D [5] and its complications [6]. However, most PRS have been developed from individuals of European ancestry, raising concerns about their generalizability across diverse populations [7–12]. Key challenges include differences in genetic architecture, sample size limitations, population admixture, and the underrepresentation of non-European groups in genetic databases. While creating PRS tailored to diverse ethnic groups is theoretically ideal, it is currently impractical due to data scarcity and the high costs associated with large-scale genome-wide association studies (GWAS) in underrepresented populations. Therefore, alternative strategies are needed to improve the transferability of existing PRS models, particularly those developed in European cohorts, to other ancestral groups.

We previously developed a multi-polygenic risk score (multiPRS) combining ten weighted PRS (wPRS) for outcomes and risk factors associated to T2D[6]. The multiPRS stratified T2D patients according to risk of cardiovascular and renal complications. Our study also demonstrated that participants identified at high-risk by our multiPRS were those who benefited most from the intensive therapy administered in ADVANCE trial [6,13]. The predictive model was initially developed in individuals with T2D of European descent and its performance in other populations is unknown. Here, we assessed whether this model could be adapted to accurately predict diabetes complications in two additional major ethnic groups. We compared different machine learning prediction models, including techniques tailored for imbalanced datasets.

Beyond transferability issues, there is increasing recognition that clinical prediction models must provide not only accurate predictions but also reliable estimates of uncertainty, as poorly calibrated predictions may lead to inappropriate clinical decisions [14].

Mondrian Cross-Conformal Prediction (MCCP) has gained attention in biomedical and genomic modeling for its ability to quantify prediction uncertainty, particularly in imbalanced datasets [15–19].

In this study, we address a major limitation of polygenic risk scores, namely their reduced transferability across ancestries, by leveraging MCCP as an uncertainty-aware prediction framework. We evaluated whether uncertainty-aware prediction, implemented through MCCP, improves the performance and reliability of polygenic risk score-based prediction of nephropathy (Low eGFR), stroke, and myocardial infarction (MI) across European (WB), South Asian (SA), and African/Caribbean (AFR) ancestries in individuals with type 2 diabetes, using data from the ADVANCE trial and the UK Biobank, compared with logistic regression. Beyond overall accuracy, we assess whether MCCP provides valid control of the error rate at a prespecified level and identifies individuals for whom no reliable prediction can be made properties not available from standard probability-based stratification.

## Results

The baseline characteristics of 17,574 individuals with T2D of self-declared White British, 1,145 self-declared South-Asian, and 749 self-declared African/Caribbean ethnicity, all validated by genetic ancestry grouping (Field ID 22006), from the UK Biobank are shown in Table 1. Compared to the two other ethnic groups, WB were slightly older and had diabetes at an older age resulting in a slightly shorter diabetes duration. Their risk factors such as HbA1c levels, blood pressure and BMI were only slightly different. The higher prevalence of macroalbuminuria in South Asian and African participants compared with White British individuals is consistent with both an earlier onset of type 2 diabetes, implying longer cumulative exposure to hyperglycaemia, and a higher susceptibility to diabetic nephropathy reported in these ethnic groups in the literature [20,21]. Stroke to myocardial infarction ratio was close to one in AFR group while the other ethnic groups had a higher frequency of myocardial infarction compared to stroke (Table 1). While statistical differences exist, their clinical effect sizes are modest.

**Table 1:** Characteristics of participants with T2D of White British (WB), South Asian (SA), and African (AFR) descent from UK Biobank.n, number of patients; yr, year; SD, standard deviation; BMI, body mass index; HbA1c, glycated hemoglobin; SBP, systolic blood pressure; DBP, diastolic blood pressure; UKBB, United Kingdom Biobank; MI, myocardial infarction; Low eGFR, estimated glomerular filtration rate based on the CKD-EPI 2009 formula (<60 ml/min/1.73m²) [22].

	UK Biobank (n = 19,468)			P value		
Characteristics	WB (n = 17,574)	SA (n = 1,145)	AFR (n = 749)	WB vs SA	WB vs AFR	SA vs AFR
Age, mean years (SD)	60.8 (6.6)	57.9 (7.5)	56.9 (8.2)	<0.001	<0.001	0.014
Female, n (%)	6,273 (35.7)	385 (33.6)	374 (49.9)	0.156	<0.001	<0.001
Diagnosis age, mean years (SD)	54.6 (8.4)	50.2 (8.4)	49.9 (8.9)	<0.001	<0.001	0.272
Diabetes duration, mean years (SD)	6.2 (6.1)	7.7 (6.7)	7.0 (6.7)	<0.001	0.002	0.004
BMI, mean kg/m² (SD)	31.8 (5.8)	28.9 (5.0)	31.7 (6.0)	<0.001	0.215	<0.001
HbA1c, mean % (SD)	6.9 (1.2)	7.2 (1.3)	7.3 (1.7)	<0.001	<0.001	0.717
SBP, mean mmHg (SD)	144 (18)	141 (18)	144 (20)	<0.001	0.116	0.004
DBP, mean mmHg (SD)	82 (10)	82 (10)	84 (11)	0.280	<0.001	<0.001
eGFR, ml/min/1.73m², median (IQR)	90.7 (79–98)	95.2 (84–103)	98.7 (82–111)	<0.001	<0.001	<0.001
Low eGFR, n (%)	1,124 (6.7)	68 (6.3)	47 (6.6)	0.611	0.904	0.810
Macroalbuminuria, n (%)	305 (4.4)	34 (8.3)	27 (8.3)	<0.001	0.001	0.986
Stroke, n (%)	1,264 (7.2)	68 (5.9)	43 (5.7)	0.110	0.132	0.858
MI, n (%)	2,383 (13.6)	188 (16.4)	50 (6.7)	<0.001	<0.001	<0.001

We evaluated the potential of various statistical and machine learning methods to predict the risk of stroke, MI and Low eGFR in patients with T2D. We used the ADVANCE cohort as the training cohort and the three UK Biobank populations to test the models. The input features were the 10 PRS, the age at the onset of diabetes, its duration, sex and 4 PC of genetic ancestry. The performance metric considered in these analyses was the area under the ROC curve (AUROC). Linear discriminant analysis (LDA), ridge classifier and logistic regression (LR) performed the best, with similar AUROC between the three ethnic groups (S1 Fig). The superior performance achieved by these three statistical methods is likely due to the underlying structure of the input data. Linear methods remain the most appropriate for this type of structure. Their better performance can also be attributed to the greater ability of linear models to generalize with smaller datasets, unlike other classification methods that often require larger training cohorts, particularly due to their need for extensive tuning. We therefore continued to use LR to predict the three main diabetes outcomes in the three ethnic groups.

For comparison with MCCP, we used logistic regression–based stratification (LR-SP), in which individuals were selected from the top and bottom tails of the out-of-sample predicted risk distribution, obtained from a model using the same predictors as MCCP, using symmetric quantiles to match MCCP coverage. These quantiles are reported as top/bottom percentages.

To disentangle the effects of method, ancestry, and sample size, we evaluated three complementary settings. First, in the ADVANCE-trained analyses, the model was trained in the European-ancestry ADVANCE cohort and tested separately in the WB, SA, and AFR UK Biobank populations. Second, in the WB-trained analyses, the model was trained directly in the UK Biobank WB population and tested in the SA and AFR populations, thereby removing cross-cohort differences and isolating the ancestry component. Third, in the sample-size-matched analyses, the WB and SA populations were downsampled to the AFR sample size (with the AFR population serving as the unchanged reference), to test whether the differences observed between populations were driven by sample size. In every setting, MCCP was compared with coverage-matched LR-SP across all phenotypes.

### Performance of MCCP and LR-SP in the ADVANCE-trained model tested in the three UK Biobank populations (WB, SA, and AFR)

**Fig 1:**
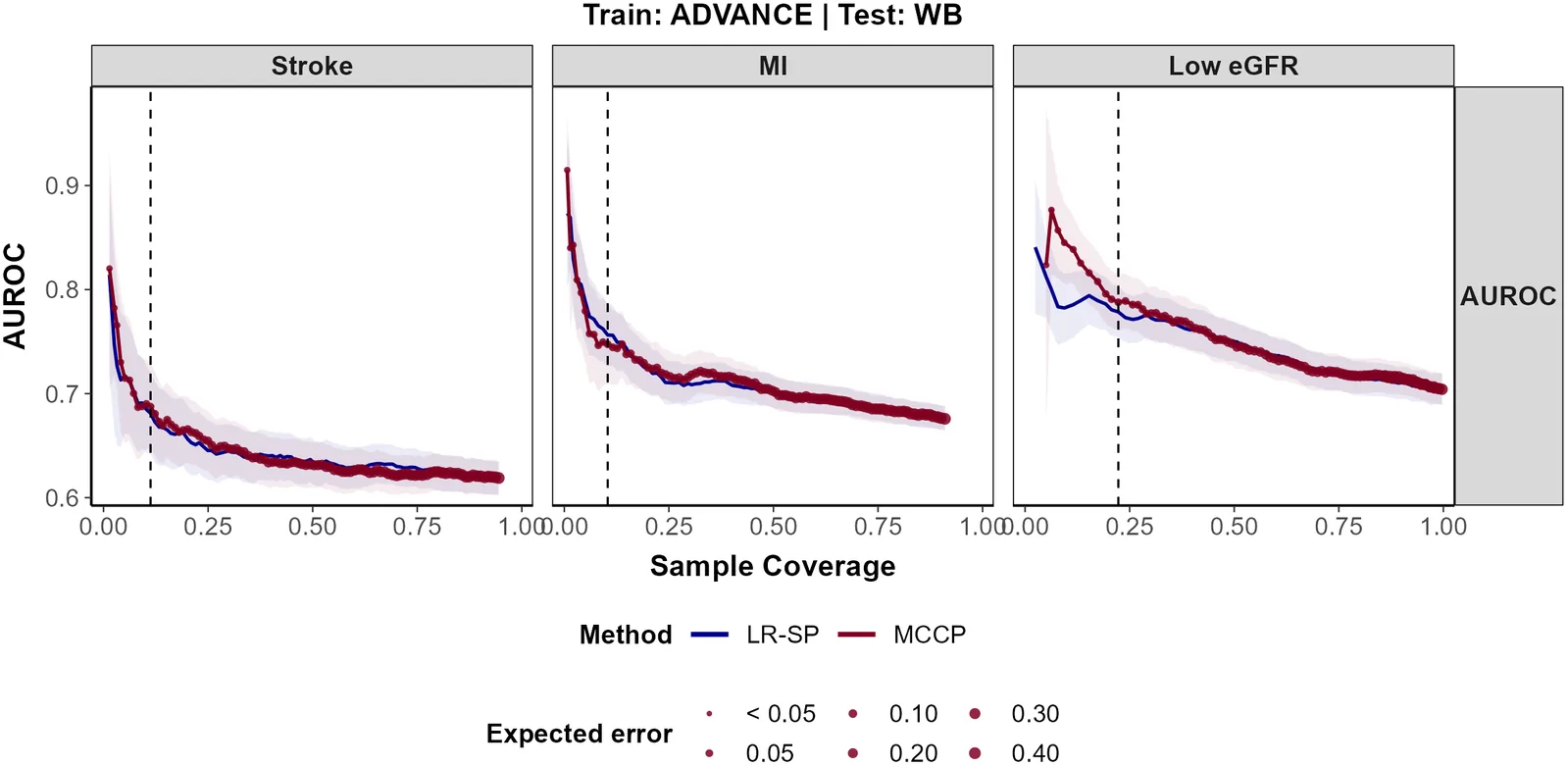
AUROC of MCCP versus LR-SP for stroke, MI, and Low eGFR in the ADVANCE-trained model tested in WB. The model was trained on ADVANCE and evaluated in the WB population, and included ten weighted polygenic risk scores, sex, age at diagnosis, diabetes duration, and PC1–PC4. The x-axis represents sample coverage. For LR-SP, coverage corresponds to the proportion of individuals selected from the top and bottom of the predicted probability distribution using symmetric quantiles matched to MCCP coverage at each error level. For MCCP, coverage refers to the proportion of individuals receiving a singleton prediction, with point size indicating the expected error α (up to 0.40). The dashed vertical line indicates the MCCP operating point closest to an expected error of α ≈ 0.05. Solid lines and shaded areas indicate the AUROC and its 95% confidence interval.

In the WB population (Tables 2 and S2, Figs 1 and S2 Figs), at an error rate of α ≈ 0.05, MCCP issued confident predictions for 10.3% of individuals for stroke, 10.4% for MI, and 20.7% for Low eGFR. Compared with coverage-matched LR-SP (top/bottom 5.1%, 5.2%, and 10.3%, respectively), the two methods showed comparable discrimination: stroke (MCCP: 0.69, 95% CI: 0.65–0.73; LR-SP: 0.68, 95% CI: 0.64–0.73), MI (0.75, 95% CI: 0.71–0.79 vs. 0.76, 95% CI: 0.73–0.79), and Low eGFR (0.79, 95% CI: 0.76–0.82 vs. 0.78, 95% CI: 0.76–0.81). NPV remained high for both methods (≥ 0.95), and PPV and recall were similar.

**Table 2:** Performance of MCCP and LR-SP in the ADVANCE-trained model tested in WB.

Phenotype	Method	Coverage (%)	Expected error (alpha)	Cases	Controls	Total	AUROC (95% CI)	PPV (95% CI)	NPV (95% CI)	Recall (95% CI)
Stroke	MCCP	10.3	0.047	130	1672	1802	0.69 (0.65-0.73)	0.11 (0.1-0.16)	0.98 (0.96-0.99)	0.85 (0.56-0.93)
Stroke	LR-SP	Top 5.1/ Bottom 5.1	–	140	1662	1802	0.68 (0.64-0.73)	0.13 (0.11-0.14)	0.97 (0.96-0.98)	0.78 (0.66-0.86)
Stroke	MCCP	94.5	0.4	1194	15411	16605	0.62 (0.6-0.64)	0.1 (0.09-0.11)	0.95 (0.95-0.96)	0.64 (0.47-0.68)
Stroke	LR-SP	Top 47.2/ Bottom 47.2	–	1193	15413	16606	0.62 (0.6-0.63)	0.1 (0.09-0.12)	0.95 (0.94-0.96)	0.63 (0.43-0.72)
MI	MCCP	10.4	0.052	176	1647	1823	0.75 (0.71-0.79)	0.22 (0.17-0.26)	0.95 (0.94-0.97)	0.66 (0.56-0.79)
MI	LR-SP	Top 5.2/ Bottom 5.2	–	238	1586	1824	0.76 (0.73-0.79)	0.26 (0.22-0.3)	0.95 (0.94-0.97)	0.78 (0.68-0.87)
MI	MCCP	90.1	0.396	2092	13737	15829	0.68 (0.66-0.69)	0.2 (0.19-0.21)	0.93 (0.92-0.94)	0.72 (0.66-0.76)
MI	LR-SP	Top 45/ Bottom 45	–	2130	13700	15830	0.68 (0.67-0.69)	0.2 (0.2-0.21)	0.93 (0.92-0.93)	0.73 (0.66-0.76)
Low eGFR	MCCP	20.7	0.045	207	3260	3467	0.79 (0.76-0.82)	0.14 (0.13-0.16)	0.98 (0.98-0.99)	0.82 (0.75-0.87)
Low eGFR	LR-SP	Top 10.3/ Bottom 10.3	–	233	3235	3468	0.78 (0.76-0.81)	0.14 (0.12-0.18)	0.98 (0.97-0.99)	0.86 (0.71-0.93)
Low eGFR	MCCP	99.7	0.348	1079	15642	16721	0.7 (0.69-0.72)	0.11 (0.1-0.12)	0.97 (0.96-0.98)	0.73 (0.61-0.82)
Low eGFR	LR-SP	Top 49.8/ Bottom 49.8	–	1084	15638	16722	0.7 (0.69-0.72)	0.11 (0.1-0.12)	0.97 (0.96-0.98)	0.74 (0.62-0.82)

Method indicates MCCP or LR-SP. The model included ten weighted polygenic risk scores, sex, age at diagnosis, diabetes duration, and PC1–PC4. For MCCP, coverage corresponds to the proportion of individuals receiving a singleton prediction at the specified expected error level α. For LR-SP, coverage was matched to MCCP by selecting individuals from the top and bottom tails of the predicted risk distribution. α denotes the expected MCCP error rate and is not applicable to LR-SP; values correspond to the closest evaluable operating point. AUROC, area under the receiver operating characteristic curve; PPV, positive predictive value; NPV, negative predictive value; Recall, sensitivity among cases included in the predicted subset.

This equivalence held at high coverage: stroke (MCCP: 0.62, 95% CI: 0.60–0.64 at 94.5% coverage, α ≈ 0.40; LR-SP: 0.62, 95% CI: 0.60–0.63), MI (0.68, 95% CI: 0.66–0.69 at 90.1%, α ≈ 0.40 vs. 0.68, 95% CI: 0.67–0.69), and Low eGFR (0.70, 95% CI: 0.69–0.72 at 99.7%, α ≈ 0.35 vs. 0.70, 95% CI: 0.69–0.72).

**Fig 2:**
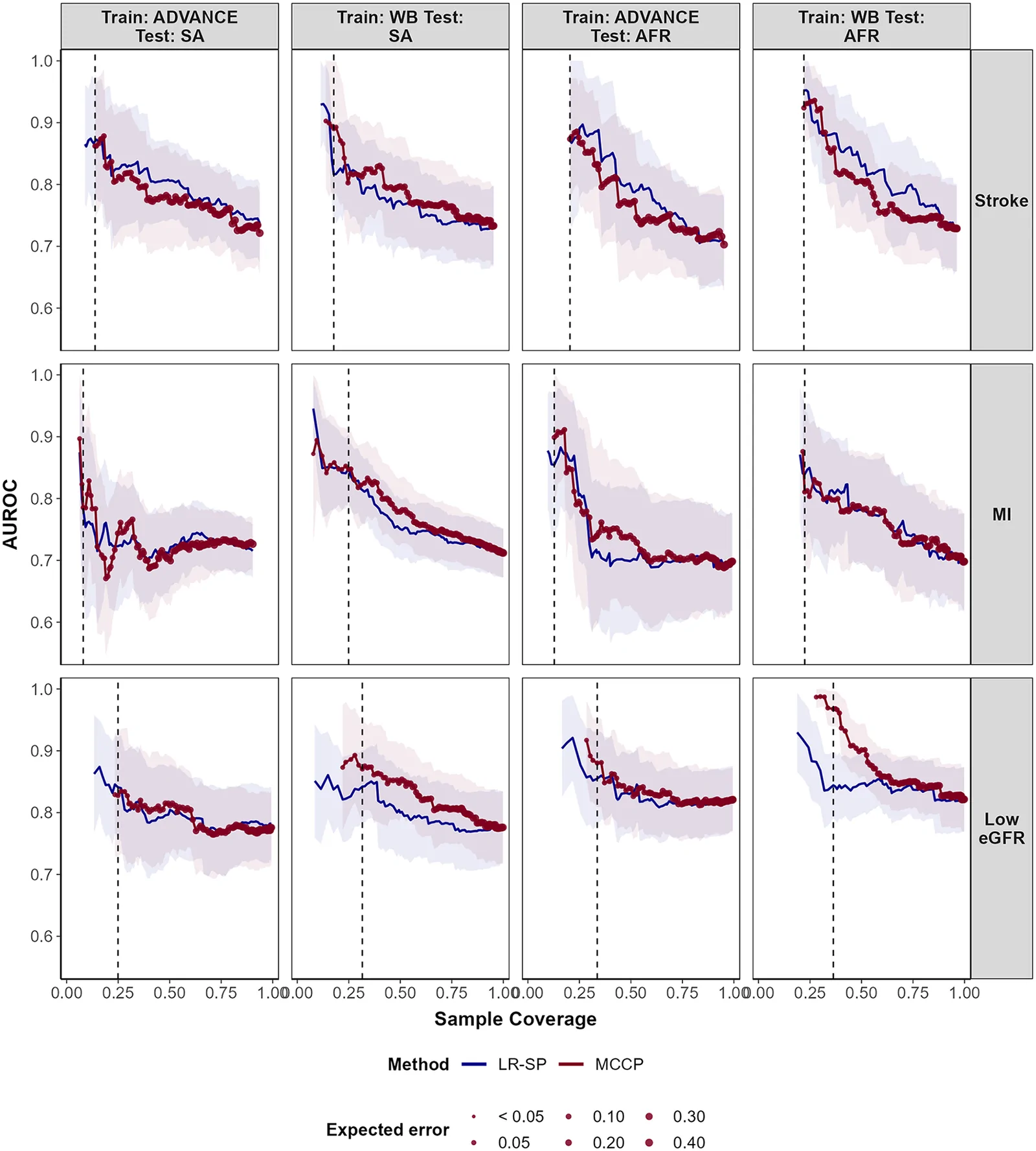
AUROC of MCCP versus LR-SP for stroke, MI and Low eGFR prediction across SA and AFR populations. See Fig 1 for legend and axis details. Models were trained on ADVANCE or WB.

In the SA population (Tables 3 and S3–S5, Figs 2 and S3–S5), at the lowest evaluated error rates, MCCP issued confident predictions for 13.8% of individuals for stroke (α ≈ 0.082), 10.1% for MI (α ≈ 0.058), and 23.4% for Low eGFR (α ≈ 0.046). Discrimination was comparable to coverage-matched LR-SP, corresponding to the top and bottom 6.9%, 5.1%, and 11.8% of the predicted probability distributions, respectively: stroke (MCCP: 0.86, 95% CI: 0.75–0.98; LR-SP: 0.87, 95% CI: 0.78–0.96), MI (0.81, 95% CI: 0.67–0.94 vs. 0.76, 95% CI: 0.61–0.92), and Low eGFR (0.83, 95% CI: 0.73–0.93 vs. 0.85, 95% CI: 0.77–0.92). Comparable discrimination was also observed at high coverage: stroke (MCCP: 0.72, 95% CI: 0.66–0.79 at 93.7% coverage, α ≈ 0.40; LR-SP: 0.74, 95% CI: 0.67–0.80), MI (0.73, 95% CI: 0.68–0.77 at 90.3% coverage, α ≈ 0.40 vs. 0.72, 95% CI: 0.67–0.76), and Low eGFR (0.77, 95% CI: 0.71–0.83 at 89.8% coverage, α ≈ 0.40 vs. 0.79, 95% CI: 0.72–0.85).

**Table 3:** Performance of MCCP and LR-SP for stroke, MI and Low eGFR prediction across SA and AFR populations.

Training population	Testing population	Phenotype	Method	Coverage (%)	Expected error (alpha)	Cases	Controls	Total	AUROC (95% CI)	PPV (95% CI)	NPV (95% CI)	Recall (95% CI)
ADVANCE	SA	Stroke	MCCP	13.8	0.082	10	148	158	0.86 (0.75-0.98)	0.32 (0.14-0.5)	0.99 (0.97-1)	0.8 (0.6-1)
ADVANCE	SA	Stroke	LR-SP	Top 6.9/ Bottom 6.9	–	13	145	158	0.87 (0.78-0.96)	0.31 (0.2-0.59)	0.99 (0.97-1)	0.92 (0.69-1)
ADVANCE	SA	Stroke	MCCP	93.7	0.4	65	1008	1073	0.72 (0.66-0.79)	0.12 (0.09-0.26)	0.97 (0.96-0.99)	0.72 (0.43-0.89)
ADVANCE	SA	Stroke	LR-SP	Top 46.9/ Bottom 46.9	–	65	1009	1074	0.74 (0.67-0.8)	0.15 (0.1-0.26)	0.97 (0.96-0.99)	0.66 (0.45-0.91)
WB	SA	Stroke	MCCP	21	0.057	17	223	240	0.87 (0.77-0.98)	0.28 (0.16-0.67)	0.99 (0.97-1)	0.88 (0.65-1)
WB	SA	Stroke	LR-SP	Top 10.5/ Bottom 10.5	–	19	221	240	0.83 (0.75-0.9)	0.22 (0.14-0.36)	0.99 (0.97-1)	0.89 (0.68-1)
WB	SA	Stroke	MCCP	95.3	0.4	64	1027	1091	0.73 (0.67-0.8)	0.17 (0.09-0.25)	0.97 (0.96-0.99)	0.59 (0.44-0.92)
WB	SA	Stroke	LR-SP	Top 47.7/ Bottom 47.7	–	65	1027	1092	0.73 (0.67-0.8)	0.12 (0.09-0.23)	0.98 (0.96-0.99)	0.75 (0.46-0.89)
ADVANCE	AFR	Stroke	MCCP	20.4	0.117	10	143	153	0.87 (0.78-0.97)	0.2 (0.13-0.8)	1 (0.98-1)	1 (0.7-1)
ADVANCE	AFR	Stroke	LR-SP	Top 10.3/ Bottom 10.3	–	10	144	154	0.87 (0.74-1)	0.4 (0.13-0.75)	0.99 (0.97-1)	0.8 (0.6-1)
ADVANCE	AFR	Stroke	MCCP	95.2	0.4	42	671	713	0.7 (0.63-0.78)	0.1 (0.09-0.18)	0.98 (0.96-0.99)	0.86 (0.52-0.95)
ADVANCE	AFR	Stroke	LR-SP	Top 47.7/ Bottom 47.7	–	42	672	714	0.71 (0.64-0.78)	0.1 (0.08-0.18)	0.98 (0.96-1)	0.83 (0.5-0.98)
WB	AFR	Stroke	MCCP	22	0.065	10	155	165	0.92 (0.86-0.99)	0.23 (0.17-0.75)	1 (0.99-1)	1 (0.8-1)
WB	AFR	Stroke	LR-SP	Top 11.1/ Bottom 11.1	–	10	156	166	0.95 (0.9-1)	0.36 (0.18-0.82)	1 (0.99-1)	1 (0.8-1)
WB	AFR	Stroke	MCCP	96.3	0.4	40	681	721	0.73 (0.66-0.8)	0.1 (0.09-0.14)	0.99 (0.97-1)	0.88 (0.7-0.98)
WB	AFR	Stroke	LR-SP	Top 48.2/ Bottom 48.2	–	40	682	722	0.73 (0.66-0.8)	0.1 (0.08-0.19)	0.98 (0.97-1)	0.82 (0.52-1)
ADVANCE	SA	MI	MCCP	10.1	0.058	13	102	115	0.81 (0.67-0.94)	0.31 (0.22-0.69)	0.97 (0.94-1)	0.85 (0.54-1)
ADVANCE	SA	MI	LR-SP	Top 5.1/ Bottom 5.1	–	16	100	116	0.76 (0.61-0.92)	0.64 (0.3-0.92)	0.94 (0.9-0.98)	0.62 (0.38-0.88)
ADVANCE	SA	MI	MCCP	90.3	0.4	161	872	1033	0.73 (0.68-0.77)	0.28 (0.24-0.32)	0.93 (0.91-0.95)	0.72 (0.6-0.83)
ADVANCE	SA	MI	LR-SP	Top 45.2/ Bottom 45.2	–	160	874	1034	0.72 (0.67-0.76)	0.26 (0.23-0.32)	0.93 (0.91-0.95)	0.77 (0.58-0.86)
WB	SA	MI	MCCP	29.5	0.062	48	290	338	0.82 (0.75-0.89)	0.43 (0.27-0.59)	0.95 (0.92-0.98)	0.71 (0.56-0.9)
WB	SA	MI	LR-SP	Top 14.8/ Bottom 14.8	–	68	270	338	0.83 (0.78-0.88)	0.47 (0.4-0.55)	0.94 (0.92-0.97)	0.82 (0.72-0.91)
WB	SA	MI	MCCP	100	0.38	188	956	1144	0.71 (0.67-0.75)	0.27 (0.24-0.37)	0.92 (0.89-0.95)	0.75 (0.52-0.86)
WB	SA	MI	LR-SP	Top 50/ Bottom 50	–	188	956	1144	0.71 (0.67-0.75)	0.27 (0.24-0.37)	0.92 (0.89-0.95)	0.75 (0.52-0.86)
ADVANCE	AFR	MI	MCCP	12.8	0.063	10	86	96	0.9 (0.8-1)	0.53 (0.2-1)	0.99 (0.95-1)	0.9 (0.6-1)
ADVANCE	AFR	MI	LR-SP	Top 6.4/ Bottom 6.4	–	11	85	96	0.85 (0.73-0.97)	0.41 (0.22-0.7)	0.98 (0.95-1)	0.82 (0.64-1)
ADVANCE	AFR	MI	MCCP	99.2	0.4	50	693	743	0.7 (0.62-0.78)	0.14 (0.1-0.24)	0.97 (0.95-0.99)	0.66 (0.44-0.92)
ADVANCE	AFR	MI	LR-SP	Top 49.7/ Bottom 49.7	–	50	694	744	0.7 (0.62-0.78)	0.15 (0.1-0.24)	0.96 (0.95-0.99)	0.62 (0.44-0.92)
WB	AFR	MI	MCCP	21.5	0.047	10	151	161	0.88 (0.77-0.98)	0.23 (0.15-0.78)	0.99 (0.97-1)	0.9 (0.6-1)
WB	AFR	MI	LR-SP	Top 10.8/ Bottom 10.8	–	12	150	162	0.84 (0.72-0.96)	0.31 (0.13-0.62)	0.98 (0.96-1)	0.83 (0.5-1)
WB	AFR	MI	MCCP	99.9	0.376	50	698	748	0.7 (0.62-0.78)	0.15 (0.1-0.24)	0.97 (0.95-0.99)	0.64 (0.44-0.92)
WB	AFR	MI	LR-SP	Top 49.9/ Bottom 49.9	–	50	698	748	0.7 (0.62-0.78)	0.15 (0.1-0.22)	0.97 (0.95-0.99)	0.64 (0.42-0.92)
ADVANCE	SA	Low eGFR	MCCP	23.4	0.046	11	242	253	0.83 (0.73-0.93)	0.11 (0.09-0.44)	1 (0.98-1)	1 (0.64-1)
ADVANCE	SA	Low eGFR	LR-SP	Top 11.8/ Bottom 11.8	–	16	238	254	0.85 (0.77-0.92)	0.19 (0.15-0.36)	0.99 (0.98-1)	0.94 (0.75-1)
ADVANCE	SA	Low eGFR	MCCP	89.8	0.4	62	908	970	0.77 (0.71-0.83)	0.16 (0.13-0.23)	0.98 (0.97-0.99)	0.77 (0.61-0.89)
ADVANCE	SA	Low eGFR	LR-SP	Top 44.9/ Bottom 44.9	–	59	911	970	0.79 (0.72-0.85)	0.17 (0.12-0.26)	0.98 (0.97-0.99)	0.75 (0.56-0.9)
WB	SA	Low eGFR	MCCP	29.8	0.045	18	304	322	0.88 (0.8-0.95)	0.31 (0.14-0.44)	0.99 (0.98-1)	0.83 (0.67-1)
WB	SA	Low eGFR	LR-SP	Top 14.9/ Bottom 14.9	–	31	291	322	0.84 (0.78-0.9)	0.27 (0.19-0.34)	0.98 (0.97-1)	0.87 (0.74-1)
WB	SA	Low eGFR	MCCP	99.8	0.36	67	1011	1078	0.78 (0.72-0.83)	0.16 (0.12-0.19)	0.98 (0.97-0.99)	0.78 (0.64-0.9)
WB	SA	Low eGFR	LR-SP	Top 49.9/ Bottom 49.9	–	67	1011	1078	0.78 (0.72-0.83)	0.16 (0.12-0.19)	0.98 (0.97-0.99)	0.78 (0.64-0.9)
ADVANCE	AFR	Low eGFR	MCCP	30.3	0.041	13	203	216	0.89 (0.82-0.97)	0.18 (0.14-0.71)	1 (0.98-1)	1 (0.69-1)
ADVANCE	AFR	Low eGFR	LR-SP	Top 15.1/ Bottom 15.1	–	19	197	216	0.86 (0.78-0.94)	0.24 (0.16-0.73)	0.99 (0.96-1)	0.89 (0.58-1)
ADVANCE	AFR	Low eGFR	MCCP	90.3	0.4	45	599	644	0.82 (0.76-0.87)	0.17 (0.13-0.26)	0.99 (0.97-1)	0.87 (0.67-1)
ADVANCE	AFR	Low eGFR	LR-SP	Top 45.2/ Bottom 45.2	–	45	599	644	0.82 (0.76-0.87)	0.18 (0.14-0.26)	0.98 (0.97-1)	0.84 (0.67-0.96)
WB	AFR	Low eGFR	MCCP	30	0.038	10	204	214	0.99 (0.97-1)	0.43 (0.3-0.91)	1 (1-1)	1 (1-1)
WB	AFR	Low eGFR	LR-SP	Top 15/ Bottom 15	–	20	194	214	0.87 (0.81-0.93)	0.28 (0.21-0.36)	0.99 (0.98-1)	0.95 (0.85-1)
WB	AFR	Low eGFR	MCCP	99.9	0.36	47	665	712	0.82 (0.77-0.87)	0.17 (0.12-0.29)	0.99 (0.97-1)	0.85 (0.64-1)
WB	AFR	Low eGFR	LR-SP	Top 49.9/ Bottom 49.9	–	47	665	712	0.82 (0.77-0.87)	0.16 (0.12-0.28)	0.99 (0.97-1)	0.85 (0.64-1)
ADVANCE	AFR	Low eGFR (AS)	MCCP	30.3	0.041	19	197	216	0.84 (0.74-0.95)	0.38 (0.16-0.8)	0.97 (0.95-1)	0.74 (0.53-1)
ADVANCE	AFR	Low eGFR (AS)	LR-SP	Top 15.1/ Bottom 15.1	–	23	193	216	0.87 (0.81-0.94)	0.26 (0.2-0.78)	0.99 (0.96-1)	0.96 (0.65-1)
ADVANCE	AFR	Low eGFR (AS)	MCCP	90.3	0.4	58	586	644	0.77 (0.71-0.82)	0.18 (0.16-0.25)	0.98 (0.96-0.99)	0.86 (0.66-0.95)
ADVANCE	AFR	Low eGFR (AS)	LR-SP	Top 45.2/ Bottom 45.2	–	57	587	644	0.77 (0.71-0.83)	0.19 (0.16-0.23)	0.98 (0.96-0.99)	0.86 (0.72-0.96)
WB	AFR	Low eGFR (AS)	MCCP	30	0.038	11	203	214	0.95 (0.9-1)	0.28 (0.16-1)	1 (0.99-1)	1 (0.82-1)
WB	AFR	Low eGFR (AS)	LR-SP	Top 15/ Bottom 15	–	24	190	214	0.88 (0.82-0.93)	0.29 (0.24-0.46)	1 (0.97-1)	1 (0.83-1)
WB	AFR	Low eGFR (AS)	MCCP	99.9	0.36	62	650	712	0.76 (0.71-0.82)	0.18 (0.16-0.22)	0.98 (0.96-0.99)	0.85 (0.69-0.94)
WB	AFR	Low eGFR (AS)	LR-SP	Top 49.9/ Bottom 49.9	–	62	650	712	0.76 (0.71-0.82)	0.18 (0.15-0.23)	0.98 (0.96-0.99)	0.85 (0.69-0.94)

See Table 2 legend for column and abbreviation details. Training population indicates whether the model was trained in the ADVANCE or WB cohort and subsequently evaluated in SA or AFR individuals.

In the AFR population (Tables 3 and S3–S5, Figs 2 and S3–S5), at the lowest evaluated error rates, MCCP issued confident predictions for 20.4% of individuals for stroke (α ≈ 0.117), 12.8% for MI (α ≈ 0.063), and 30.3% for Low eGFR (α ≈ 0.041). The lowest evaluable error level for stroke was higher than for the other outcomes. Discrimination was comparable to coverage-matched LR-SP, corresponding to the top and bottom 10.3%, 6.4%, and 15.1% of the predicted probability distributions, respectively: stroke (MCCP: 0.87, 95% CI: 0.78–0.97; LR-SP: 0.87, 95% CI: 0.74–1.00), MI (0.90, 95% CI: 0.80–1.00 vs. 0.85, 95% CI: 0.73–0.97), and Low eGFR (0.89, 95% CI: 0.82–0.97 vs. 0.86, 95% CI: 0.78–0.94). Comparable discrimination was also observed at high coverage: stroke (MCCP: 0.70, 95% CI: 0.63–0.78 at 95.2% coverage, α ≈ 0.40; LR-SP: 0.71, 95% CI: 0.64–0.78), MI (0.70, 95% CI: 0.62–0.78 at 99.2% coverage, α ≈ 0.40 vs. 0.70, 95% CI: 0.62–0.78), and Low eGFR (0.82, 95% CI: 0.76–0.87 at 90.3% coverage, α ≈ 0.40 vs. 0.82, 95% CI: 0.76–0.87).

**Fig 3:**
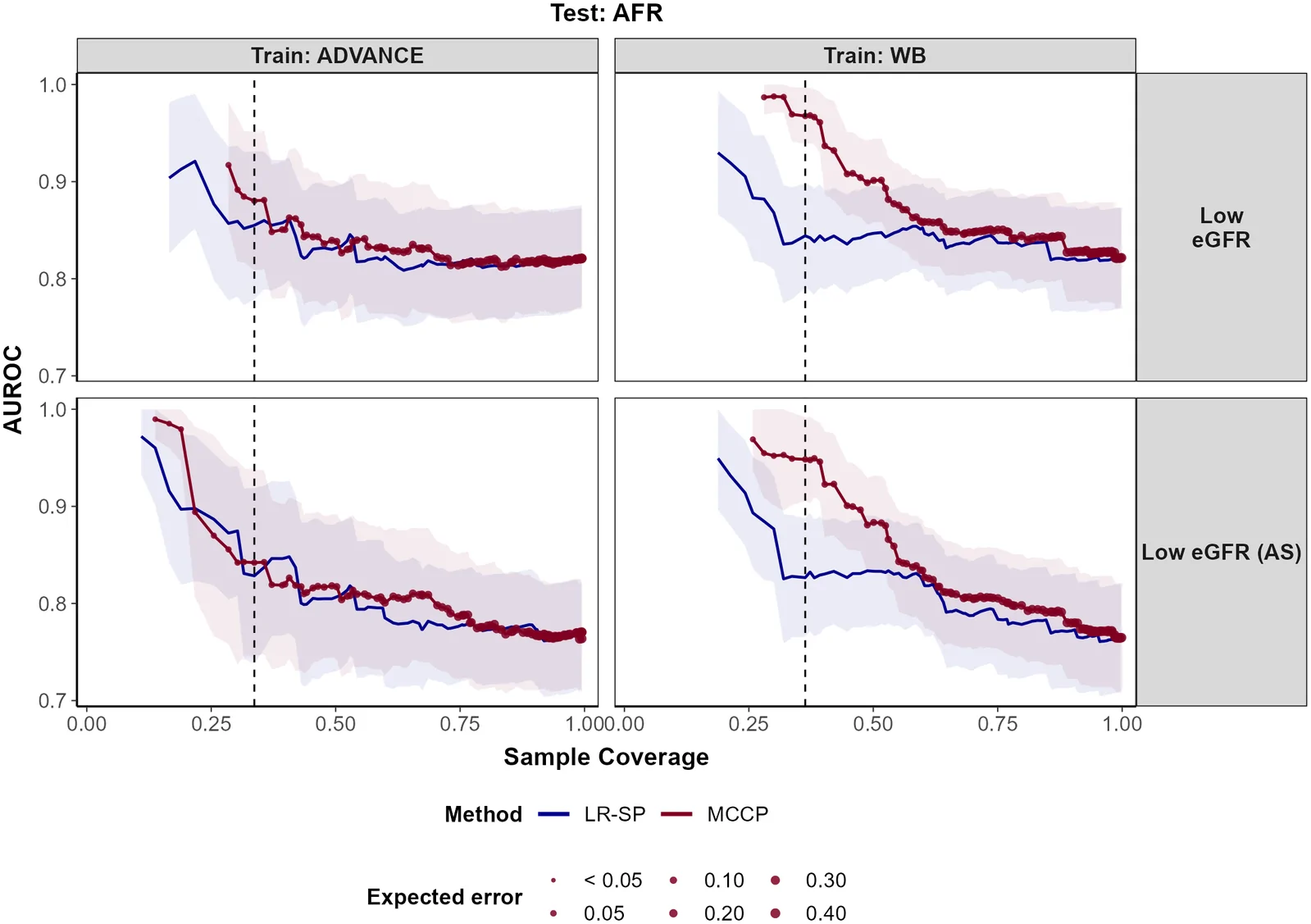
AUROC of MCCP versus LR-SP for Low eGFR with and without the race coefficient in AFR. See Fig 1 for axis details. Models were trained on ADVANCE or WB. (A) Low eGFR was defined using the CKD-EPI 2009 equation including the race coefficient [22]. (B) Low eGFR (AS) was defined using the race-free CKD-EPI 2021 equation [23].

Removing the race coefficient from the eGFR equation lowered the mean estimated eGFR in the AFR population from 95.4 ± 22.1 to 86.6 ± 19.3 mL/min/1.73 m² and increased the number of Low eGFR cases from 47 (6.6%) to 62 (8.7%), reflecting the reclassification of 15 individuals (2.1%) from control to case status (S1 Table). Despite this shift in case definition, discrimination under the race-free CKD-EPI definition remained comparable between methods: at α ≈ 0.04 and 30.3% MCCP coverage, MCCP achieved an AUROC of 0.84 (95% CI: 0.74–0.95), compared with 0.87 (95% CI: 0.81–0.94) for coverage-matched LR-SP. At 90.3% coverage and α ≈ 0.40, both methods achieved an AUROC of 0.77 (MCCP: 95% CI: 0.71–0.82; LR-SP: 95% CI: 0.71–0.83) (Fig 3 and S6, S5 Tables).

### Performance of MCCP and LR-SP in the WB-trained model tested in SA and AFR

In the SA population (Tables 3 and S3–S5, Figs 2 and S3–S5), at the lowest evaluated error rates, MCCP issued confident predictions for 21.0% of individuals for stroke (α ≈ 0.057), 29.5% for MI (α ≈ 0.062), and 29.8% for Low eGFR (α ≈ 0.045). Discrimination was comparable to coverage-matched LR-SP, corresponding to the top and bottom 10.5%, 14.8%, and 14.9% of the predicted probability distributions, respectively: stroke (MCCP: 0.87, 95% CI: 0.77–0.98; LR-SP: 0.83, 95% CI: 0.75–0.90), MI (0.82, 95% CI: 0.75–0.89 vs. 0.83, 95% CI: 0.78–0.88), and Low eGFR (0.88, 95% CI: 0.80–0.95 vs. 0.84, 95% CI: 0.78–0.90). Comparable discrimination was observed at high coverage: stroke (MCCP: 0.73, 95% CI: 0.67–0.80 at 95.3% coverage, α ≈ 0.40; LR-SP: 0.73, 95% CI: 0.67–0.80), MI (0.71, 95% CI: 0.67–0.75 at 100% coverage, α ≈ 0.38 vs. 0.71, 95% CI: 0.67–0.75), and Low eGFR (0.78, 95% CI: 0.72–0.83 at 99.8% coverage, α ≈ 0.36 vs. 0.78, 95% CI: 0.72–0.83).

In the AFR population (Tables 3 and S3–S5, Figs 2 and S3–S5), at the lowest evaluated error rates, MCCP issued confident predictions for 22.0% of individuals for stroke (α ≈ 0.065), 21.5% for MI (α ≈ 0.047), and 30.0% for Low eGFR (α ≈ 0.038). Discrimination was comparable to coverage-matched LR-SP, corresponding to the top and bottom 11.1%, 10.8%, and 15.0% of the predicted probability distributions, respectively: stroke (MCCP: 0.92, 95% CI: 0.86–0.99; LR-SP: 0.95, 95% CI: 0.90–1.00), MI (0.88, 95% CI: 0.77–0.98 vs. 0.84, 95% CI: 0.72–0.96), and Low eGFR (0.99, 95% CI: 0.97–1.00 vs. 0.87, 95% CI: 0.81–0.93). Under the race-free CKD-EPI definition, Low eGFR (AS) yielded similar results (MCCP: 0.95, 95% CI: 0.90–1.00; LR-SP: 0.88, 95% CI: 0.82–0.93). Comparable discrimination was observed at high coverage: stroke (MCCP: 0.73, 95% CI: 0.66–0.80 at 96.3% coverage, α ≈ 0.40; LR-SP: 0.73, 95% CI: 0.66–0.80), MI (0.70, 95% CI: 0.62–0.78 at 99.9% coverage, α ≈ 0.38 vs. 0.70, 95% CI: 0.62–0.78), Low eGFR (0.82, 95% CI: 0.77–0.87 at 99.9% coverage, α ≈ 0.36 vs. 0.82, 95% CI: 0.77–0.87), and Low eGFR (AS) (0.76, 95% CI: 0.71–0.82 vs. 0.76, 95% CI: 0.71–0.82) (Figs 3 and S6, S5 Table)

### Performance of MCCP and LR-SP after sample size adjustments

We uniformized population sizes by applying stratified random subsampling based on case–control status, reducing the WB and SA populations to match the AFR sample size while preserving class balance for each phenotype. The distributions of key covariates (age at diagnosis, diabetes duration, sex, and principal components, PC1–PC4) were maintained between the original and downsampled datasets. Statistical tests (Kolmogorov–Smirnov [24] for continuous variables and Fisher’s exact test [25] for categorical variables) revealed no significant differences, confirming the representativeness of the downsampled samples **(**S6 Table, S7–S12 Figs**)**.

This adjustment was intended to assess whether the observed differences among the WB, SA, and AFR populations were attributable to differences in sample size rather than methodological bias.

**Fig 4:**
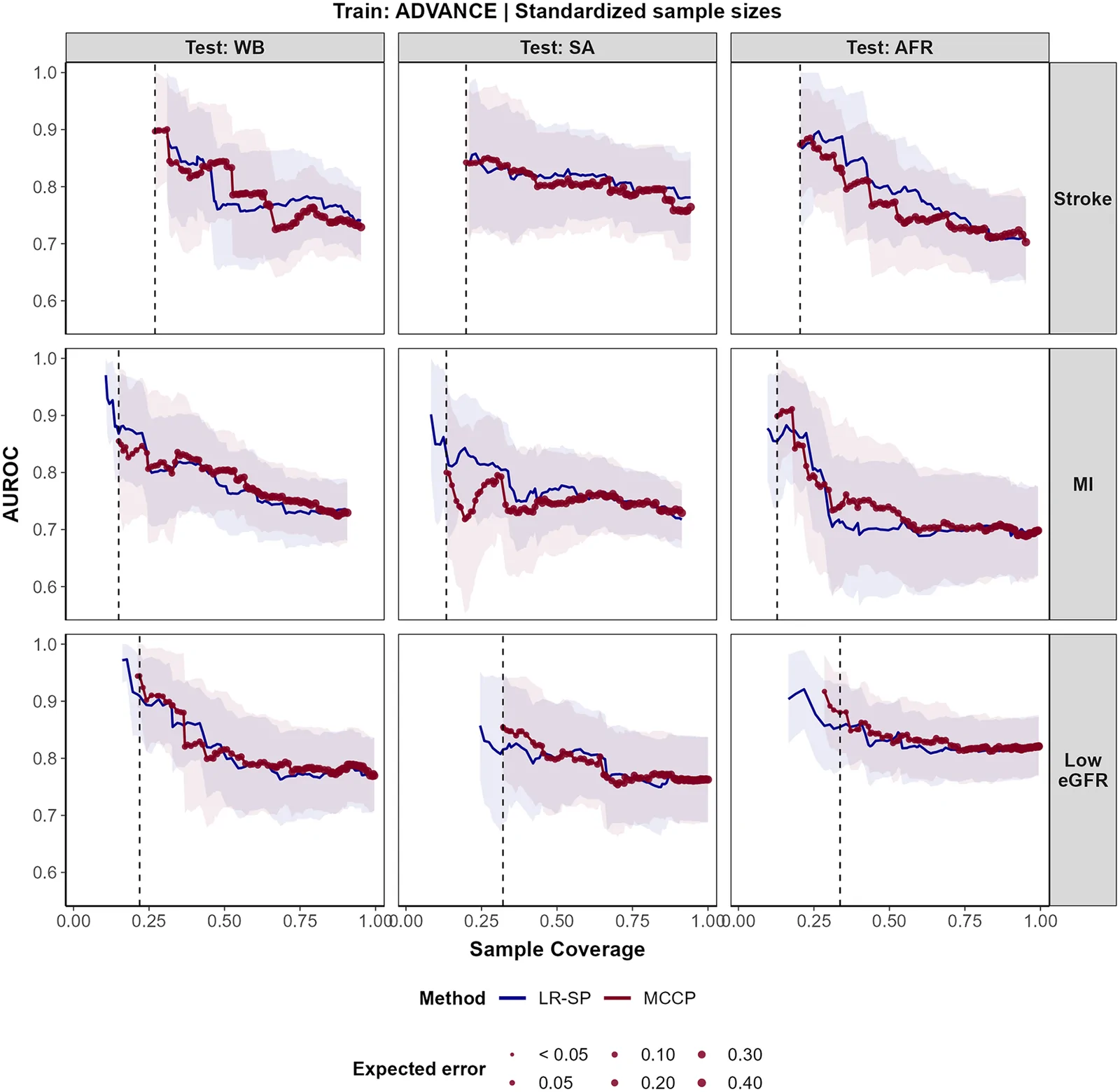
AUROC of MCCP versus LR-SP after sample size standardization. Rows show stroke, MI, and Low eGFR, and columns show the test population (WB, SA, and AFR). WB and SA were downsampled by stratified random subsampling to match the AFR sample size, whereas AFR was not downsampled and is shown as the reference population. See Fig 1 for axis details.

In the downsampled WB population (Tables 4 and S7, Figs 4 and S13–S15), at the lowest evaluated error rates, MCCP issued confident predictions for 27.0% of individuals for stroke (α ≈ 0.109), 15.0% for MI (α ≈ 0.071), and 21.2% for Low eGFR (α ≈ 0.044). The corresponding LR-SP analyses used the top and bottom 15.5%, 7.5%, and 10.7% of the predicted probability distributions, respectively. Discrimination was comparable between MCCP and LR-SP: stroke (MCCP: 0.90, 95% CI: 0.79–1.00; LR-SP: 0.89, 95% CI: 0.77–1.00), MI (0.85, 95% CI: 0.72–0.99 vs. 0.87, 95% CI: 0.78–0.96), and Low eGFR (0.94, 95% CI: 0.89–1.00 vs. 0.91, 95% CI: 0.84–0.98). Comparable discrimination was observed at high coverage: stroke (MCCP: 0.73, 95% CI: 0.67–0.79 at 95.2% coverage, α ≈ 0.40; LR-SP: 0.74, 95% CI: 0.68–0.80), MI (0.73, 95% CI: 0.68–0.78 at 89.9% coverage, α ≈ 0.40 vs. 0.74, 95% CI: 0.68–0.79), and Low eGFR (0.77, 95% CI: 0.70–0.83 at 99.6% coverage, α ≈ 0.35 vs. 0.77, 95% CI: 0.71–0.84).

**Table 4:** Performance of MCCP and LR-SP for stroke, MI, and Low eGFR after downsampling WB and SA to the AFR sample size.

Training population	Testing population	Phenotype	Method	Coverage (%)	Expected error (alpha)	Cases	Controls	Total	AUROC (95% CI)	PPV (95% CI)	NPV (95% CI)	Recall (95% CI)
ADVANCE	WB	Stroke	MCCP	27	0.109	10	192	202	0.9 (0.79-1)	0.35 (0.17-0.58)	0.99 (0.98-1)	0.9 (0.7-1)
ADVANCE	WB	Stroke	LR-SP	Top 15.5/ Bottom 15.5	–	10	222	232	0.89 (0.77-1)	0.31 (0.14-0.57)	0.99 (0.98-1)	0.9 (0.6-1)
ADVANCE	WB	Stroke	MCCP	95.2	0.4	50	663	713	0.73 (0.67-0.79)	0.14 (0.12-0.17)	0.98 (0.96-0.99)	0.82 (0.68-0.94)
ADVANCE	WB	Stroke	LR-SP	Top 47.7/ Bottom 47.7	–	47	667	714	0.74 (0.68-0.8)	0.14 (0.11-0.17)	0.98 (0.97-0.99)	0.79 (0.66-0.94)
ADVANCE	SA	Stroke	MCCP	19.9	0.109	10	139	149	0.84 (0.72-0.97)	0.31 (0.12-0.56)	0.98 (0.97-1)	0.8 (0.6-1)
ADVANCE	SA	Stroke	LR-SP	Top 10.5/ Bottom 10.5	–	10	148	158	0.84 (0.7-0.98)	0.33 (0.1-0.64)	0.98 (0.97-1)	0.8 (0.5-1)
ADVANCE	SA	Stroke	MCCP	94.3	0.4	39	667	706	0.76 (0.68-0.85)	0.14 (0.1-0.29)	0.98 (0.97-0.99)	0.77 (0.51-0.92)
ADVANCE	SA	Stroke	LR-SP	Top 47.1/ Bottom 47.1	–	39	667	706	0.78 (0.7-0.86)	0.15 (0.1-0.29)	0.98 (0.97-0.99)	0.77 (0.51-0.95)
ADVANCE	AFR	Stroke	MCCP	20.4	0.117	10	143	153	0.87 (0.78-0.97)	0.2 (0.13-0.8)	1 (0.98-1)	1 (0.7-1)
ADVANCE	AFR	Stroke	LR-SP	Top 10.3/ Bottom 10.3	–	10	144	154	0.87 (0.74-1)	0.4 (0.13-0.75)	0.99 (0.97-1)	0.8 (0.6-1)
ADVANCE	AFR	Stroke	MCCP	95.2	0.4	42	671	713	0.7 (0.63-0.78)	0.1 (0.09-0.18)	0.98 (0.96-0.99)	0.86 (0.52-0.95)
ADVANCE	AFR	Stroke	LR-SP	Top 47.7/ Bottom 47.7	–	42	672	714	0.71 (0.64-0.78)	0.1 (0.08-0.18)	0.98 (0.96-1)	0.83 (0.5-0.98)
ADVANCE	WB	MI	MCCP	15	0.071	10	102	112	0.85 (0.72-0.99)	0.5 (0.16-0.86)	0.98 (0.95-1)	0.8 (0.5-1)
ADVANCE	WB	MI	LR-SP	Top 7.5/ Bottom 7.5	–	15	97	112	0.87 (0.78-0.96)	0.45 (0.24-0.75)	0.98 (0.94-1)	0.87 (0.6-1)
ADVANCE	WB	MI	MCCP	89.9	0.396	84	589	673	0.73 (0.68-0.78)	0.23 (0.18-0.29)	0.95 (0.93-0.98)	0.75 (0.58-0.94)
ADVANCE	WB	MI	LR-SP	Top 45/ Bottom 45	–	87	587	674	0.74 (0.68-0.79)	0.25 (0.21-0.3)	0.95 (0.93-0.97)	0.75 (0.61-0.86)
ADVANCE	SA	MI	MCCP	13.4	0.071	10	90	100	0.8 (0.64-0.96)	0.38 (0.16-0.89)	0.97 (0.94-1)	0.8 (0.4-1)
ADVANCE	SA	MI	LR-SP	Top 6.7/ Bottom 6.7	–	16	84	100	0.83 (0.7-0.97)	0.61 (0.39-1)	0.95 (0.91-0.99)	0.75 (0.56-0.94)
ADVANCE	SA	MI	MCCP	90.5	0.396	107	571	678	0.73 (0.68-0.78)	0.31 (0.24-0.37)	0.93 (0.91-0.96)	0.71 (0.57-0.89)
ADVANCE	SA	MI	LR-SP	Top 45.3/ Bottom 45.3	–	107	571	678	0.72 (0.67-0.77)	0.29 (0.24-0.35)	0.93 (0.91-0.96)	0.73 (0.59-0.88)
ADVANCE	AFR	MI	MCCP	12.8	0.063	10	86	96	0.9 (0.8-1)	0.53 (0.2-1)	0.99 (0.95-1)	0.9 (0.6-1)
ADVANCE	AFR	MI	LR-SP	Top 6.4/ Bottom 6.4	–	11	85	96	0.85 (0.73-0.97)	0.41 (0.22-0.7)	0.98 (0.95-1)	0.82 (0.64-1)
ADVANCE	AFR	MI	MCCP	99.2	0.4	50	693	743	0.7 (0.62-0.78)	0.14 (0.1-0.24)	0.97 (0.95-0.99)	0.66 (0.44-0.92)
ADVANCE	AFR	MI	LR-SP	Top 49.7/ Bottom 49.7	–	50	694	744	0.7 (0.62-0.78)	0.15 (0.1-0.24)	0.96 (0.95-0.99)	0.62 (0.44-0.92)
ADVANCE	WB	Low eGFR	MCCP	21.2	0.044	10	141	151	0.94 (0.89-1)	0.4 (0.19-0.8)	1 (0.99-1)	1 (0.8-1)
ADVANCE	WB	Low eGFR	LR-SP	Top 10.7/ Bottom 10.7	–	12	140	152	0.91 (0.84-0.98)	0.25 (0.19-0.89)	1 (0.98-1)	1 (0.75-1)
ADVANCE	WB	Low eGFR	MCCP	99.6	0.349	54	656	710	0.77 (0.7-0.83)	0.17 (0.14-0.28)	0.98 (0.96-0.99)	0.8 (0.59-0.93)
ADVANCE	WB	Low eGFR	LR-SP	Top 49.8/ Bottom 49.8	–	55	655	710	0.77 (0.71-0.84)	0.2 (0.14-0.29)	0.97 (0.96-0.99)	0.76 (0.56-0.93)
ADVANCE	SA	Low eGFR	MCCP	32.1	0.067	10	219	229	0.85 (0.77-0.94)	0.13 (0.09-0.31)	1 (0.99-1)	1 (0.7-1)
ADVANCE	SA	Low eGFR	LR-SP	Top 16.1/ Bottom 16.1	–	14	216	230	0.82 (0.69-0.94)	0.23 (0.15-0.5)	0.98 (0.97-1)	0.79 (0.5-1)
ADVANCE	SA	Low eGFR	MCCP	90.3	0.396	40	604	644	0.76 (0.68-0.84)	0.13 (0.11-0.26)	0.98 (0.97-0.99)	0.82 (0.52-0.95)
ADVANCE	SA	Low eGFR	LR-SP	Top 45.2/ Bottom 45.2	–	39	605	644	0.76 (0.69-0.84)	0.14 (0.1-0.27)	0.98 (0.97-1)	0.79 (0.51-0.97)
ADVANCE	AFR	Low eGFR	MCCP	30.3	0.041	13	203	216	0.89 (0.82-0.97)	0.18 (0.14-0.71)	1 (0.98-1)	1 (0.69-1)
ADVANCE	AFR	Low eGFR	LR-SP	Top 15.1/ Bottom 15.1	–	19	197	216	0.86 (0.78-0.94)	0.24 (0.16-0.73)	0.99 (0.96-1)	0.89 (0.58-1)
ADVANCE	AFR	Low eGFR	MCCP	90.3	0.4	45	599	644	0.82 (0.76-0.87)	0.17 (0.13-0.26)	0.99 (0.97-1)	0.87 (0.67-1)
ADVANCE	AFR	Low eGFR	LR-SP	Top 45.2/ Bottom 45.2	–	45	599	644	0.82 (0.76-0.87)	0.18 (0.14-0.26)	0.98 (0.97-1)	0.84 (0.67-0.96)

See Table 2 legend for column and abbreviation details. WB and SA populations were downsampled via stratified random subsampling to match the AFR sample size, whereas the AFR population was not downsampled and is shown as the reference population.

In the downsampled SA population (Tables 4 and S7, Figs 4 and S13–S15), at the lowest evaluated error rates, MCCP issued confident predictions for 19.9% of individuals for stroke (α ≈ 0.109), 13.4% for MI (α ≈ 0.071), and 32.1% for Low eGFR (α ≈ 0.067). The corresponding LR-SP analyses used the top and bottom 10.5%, 6.7%, and 16.1% of the predicted probability distributions, respectively. Discrimination was comparable between MCCP and LR-SP: stroke (MCCP: 0.84, 95% CI: 0.72–0.97; LR-SP: 0.84, 95% CI: 0.70–0.98), MI (0.80, 95% CI: 0.64–0.96 vs. 0.83, 95% CI: 0.70–0.97), and Low eGFR (0.85, 95% CI: 0.77–0.94 vs. 0.82, 95% CI: 0.69–0.94). Comparable discrimination was observed at high coverage: stroke (MCCP: 0.76, 95% CI: 0.68–0.85 at 94.3% coverage, α ≈ 0.40; LR-SP: 0.78, 95% CI: 0.70–0.86), MI (0.73, 95% CI: 0.68–0.78 at 90.5% coverage, α ≈ 0.40 vs. 0.72, 95% CI: 0.67–0.77), and Low eGFR (0.76, 95% CI: 0.68–0.84 at 90.3% coverage, α ≈ 0.40 vs. 0.76, 95% CI: 0.69–0.84).

Because the AFR population served as the reference sample size, it was not downsampled; its results therefore remained unchanged and are presented in Table 3 and Fig 3.

## Calibration and reclassification

### Expected and observed error

**Fig 5:**
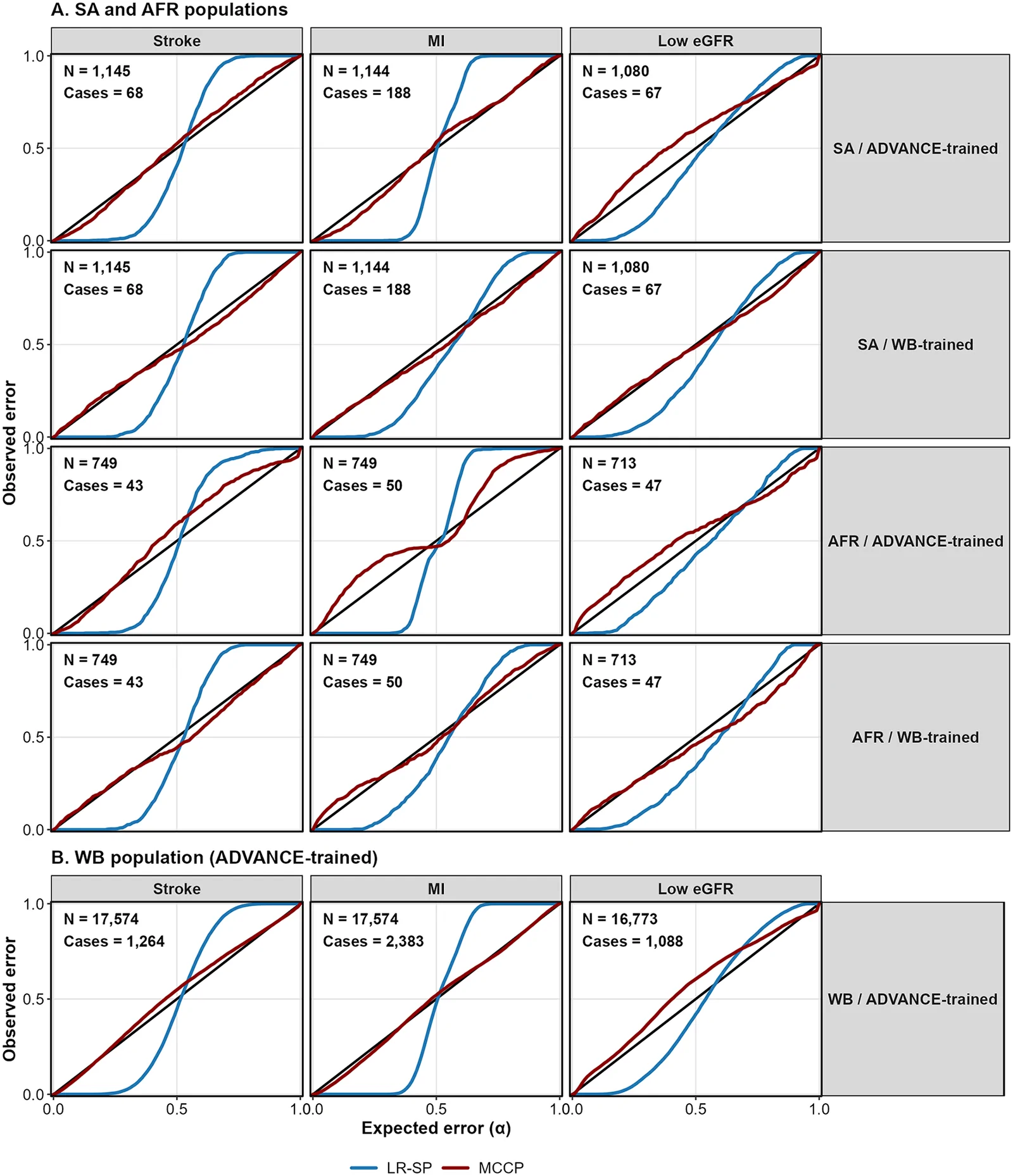
Expected versus observed error for MCCP and LR-SP across populations and training cohorts.

Expected and observed error rates were compared across the full range of α values (0–1) for MCCP and LR-SP (Fig 5). Overall, the observed-error curves for MCCP followed the diagonal more closely than those for LR-SP across most phenotype–population combinations. Agreement with the diagonal was particularly close for stroke and MI in the WB and SA populations. More pronounced departures were observed in the AFR population under the ADVANCE-trained models (Fig 5A), especially for MI and Low eGFR, for which the observed error generally exceeded the expected error over the low-to-intermediate range of α. The departure was less marked for stroke. Under the WB-trained models, MCCP remained generally close to the diagonal in both the SA and AFR populations; notably, the observed error for Low eGFR in SA closely followed the expected error (Fig 5A). In AFR, the race-free Low eGFR definition produced a pattern similar to that observed with the original definition (S16 Fig). LR-SP showed more pronounced, predominantly sigmoidal departures from the diagonal across phenotypes and populations.

For each expected error level α (0–1), the observed error is plotted against α; the diagonal line indicates perfect calibration, where observed error equals expected error. **(A)** SA and AFR populations, under both the ADVANCE-trained and WB-trained models. **(B)** WB population, under the ADVANCE-trained model. Each model included ten weighted polygenic risk scores, sex, age at diagnosis, diabetes duration, and PC1–PC4. MCCP, Mondrian Cross-Conformal Prediction; LR-SP, logistic regression–based stratification by predicted probabilities; WB, White British; SA, South Asian; AFR, African/Caribbean.

### Empirical miscoverage (MCCP)

**Fig 6:**
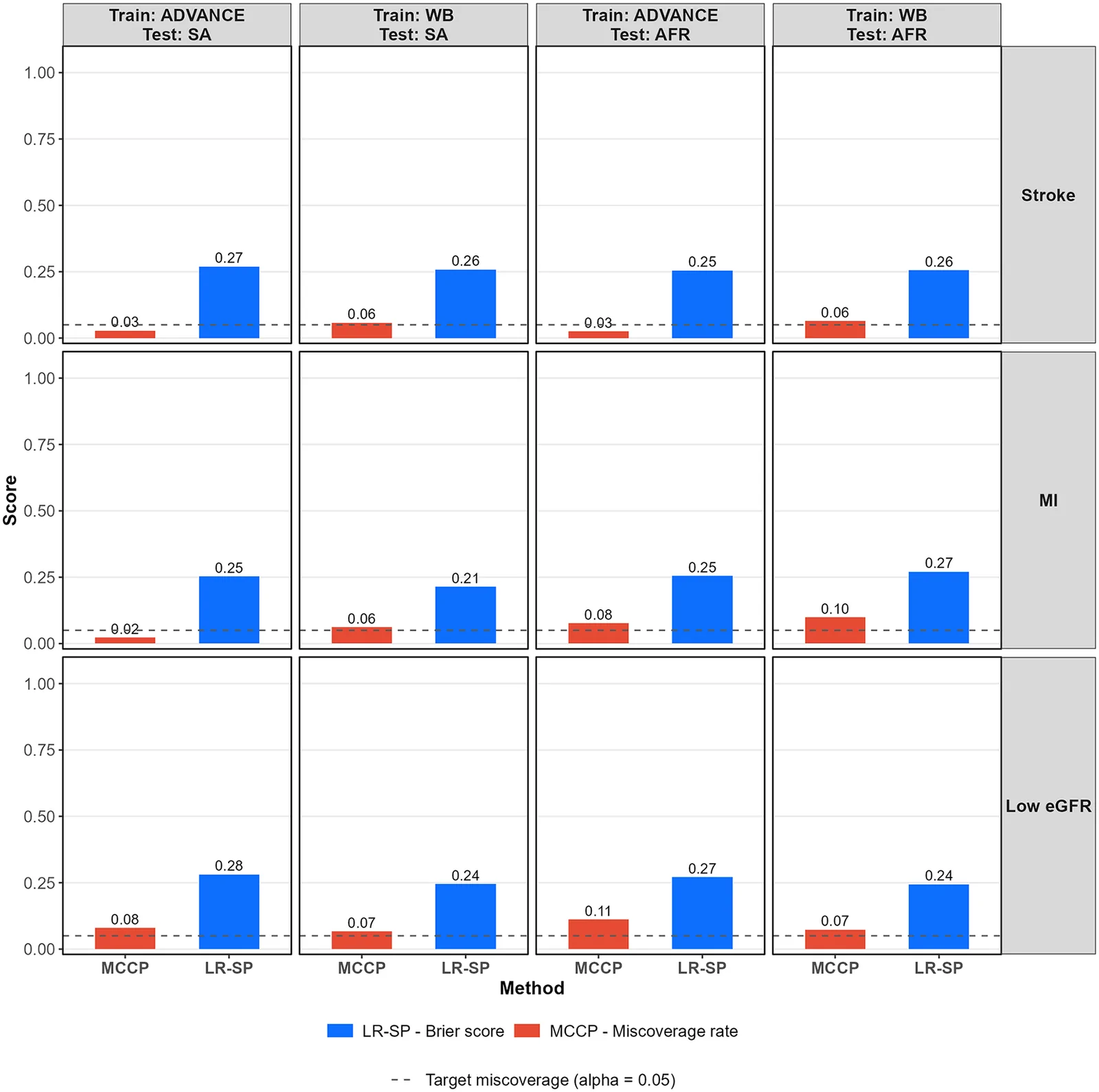
Empirical miscoverage of MCCP and Brier score of LR-SP for stroke, MI and Low eGFR in the SA and AFR populations, at α = 0.05.

**Fig 7:**
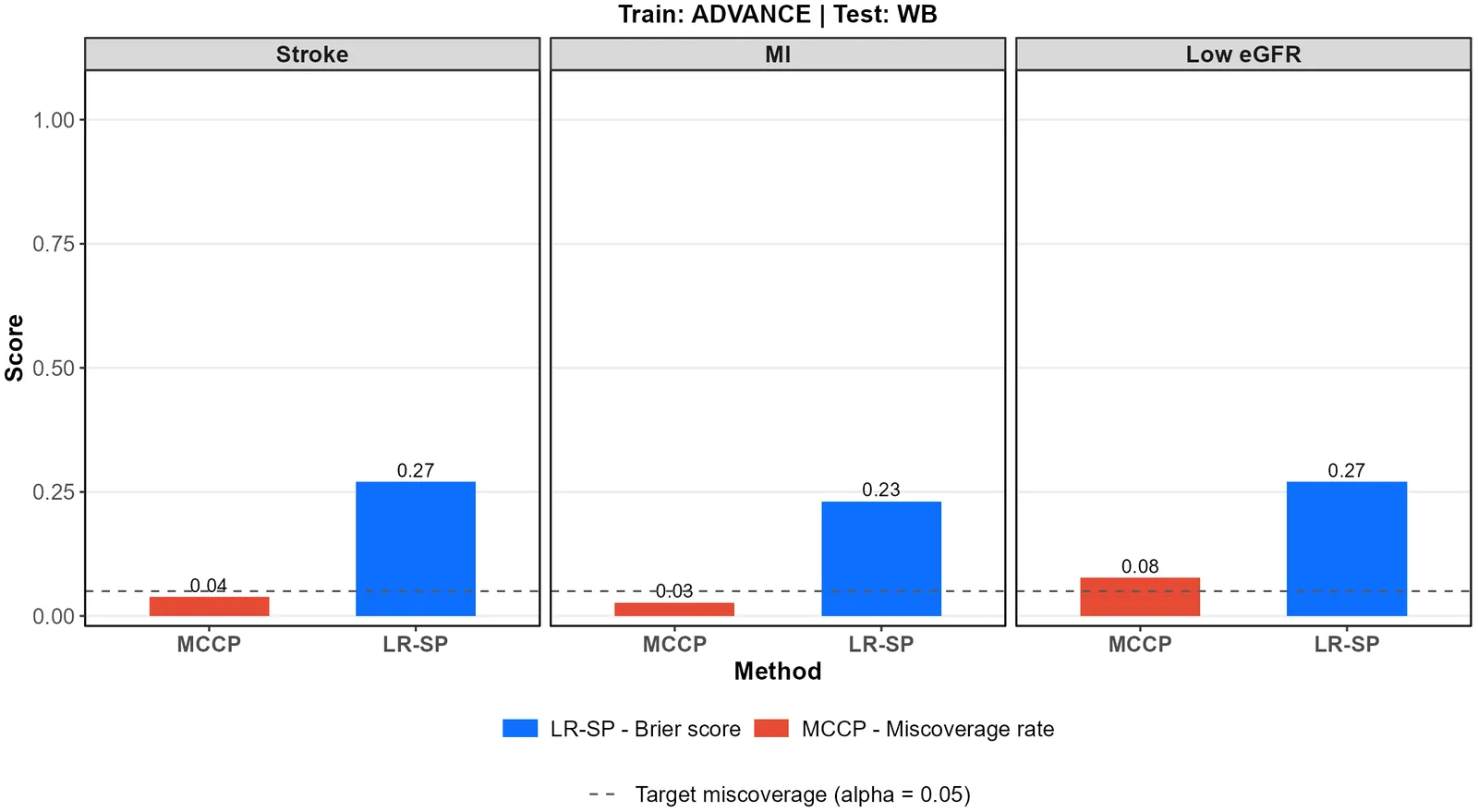
Empirical miscoverage of MCCP and Brier score of LR-SP in the WB population (ADVANCE-trained), at α = 0.05.

Calibration of MCCP was assessed using empirical miscoverage rates across the full range of α values (0.05–0.40) (Figs 6,7). At α ≈ 0.05, empirical miscoverage for stroke in the ADVANCE-trained model was 0.038, 0.028, and 0.025 in the WB, SA, and AFR populations, respectively. In the WB-trained model, the corresponding values were 0.058 and 0.064 in the SA and AFR populations. For MI, empirical miscoverage was 0.027, 0.023, and 0.076 in the WB, SA, and AFR populations, respectively, for the ADVANCE-trained model, and 0.062 and 0.099 in the SA and AFR populations for the WB-trained model. For Low eGFR, empirical miscoverage ranged from 0.067 to 0.111 across phenotype–population combinations. Similar values were observed for Low eGFR (AS), with empirical miscoverage of 0.108 in the AFR population for the ADVANCE-trained model and 0.074 in the AFR population for the WB-trained model (S17 Fig).

Bars show two distinct calibration measures, appropriate to each method: empirical miscoverage for MCCP (proportion of individuals whose true label fell outside the prediction region) and the Brier score for LR-SP at matched coverage. The two measures quantify different properties and are not directly comparable. The dashed line indicates the target error level (α = 0.05). Panels are arranged by phenotype (rows) and by training cohort and test population (columns). MCCP, Mondrian Cross-Conformal Prediction; LR-SP, logistic regression–based stratification by predicted probabilities; SA, South Asian; AFR, African/Caribbean.

Measures are shown as in Fig 6, for stroke, MI, and Low eGFR. Abbreviations as in Fig 6.

### Brier score (LR-SP)

For LR-SP, probabilistic calibration was assessed using the Brier score at MCCP-matched coverage levels (Figs 6,7). At α ≈ 0.05, Brier scores in the ADVANCE-trained model ranged from 0.231 to 0.281, with the lowest value observed for MI in the WB population and the highest value observed for Low eGFR in the SA population. In the WB-trained model, Brier scores ranged from 0.214 to 0.271, with the lowest value observed for MI in the SA population and the highest value observed for MI in the AFR population. For Low eGFR, Brier scores were broadly similar across populations and training cohorts, ranging from 0.244 to 0.281. Comparable values were observed for Low eGFR (AS) in the AFR population, with Brier scores of 0.264 and 0.239 in the ADVANCE-trained and WB-trained models, respectively (S17 Fig).

### Net reclassification improvement (NRI)

**Fig 8:**
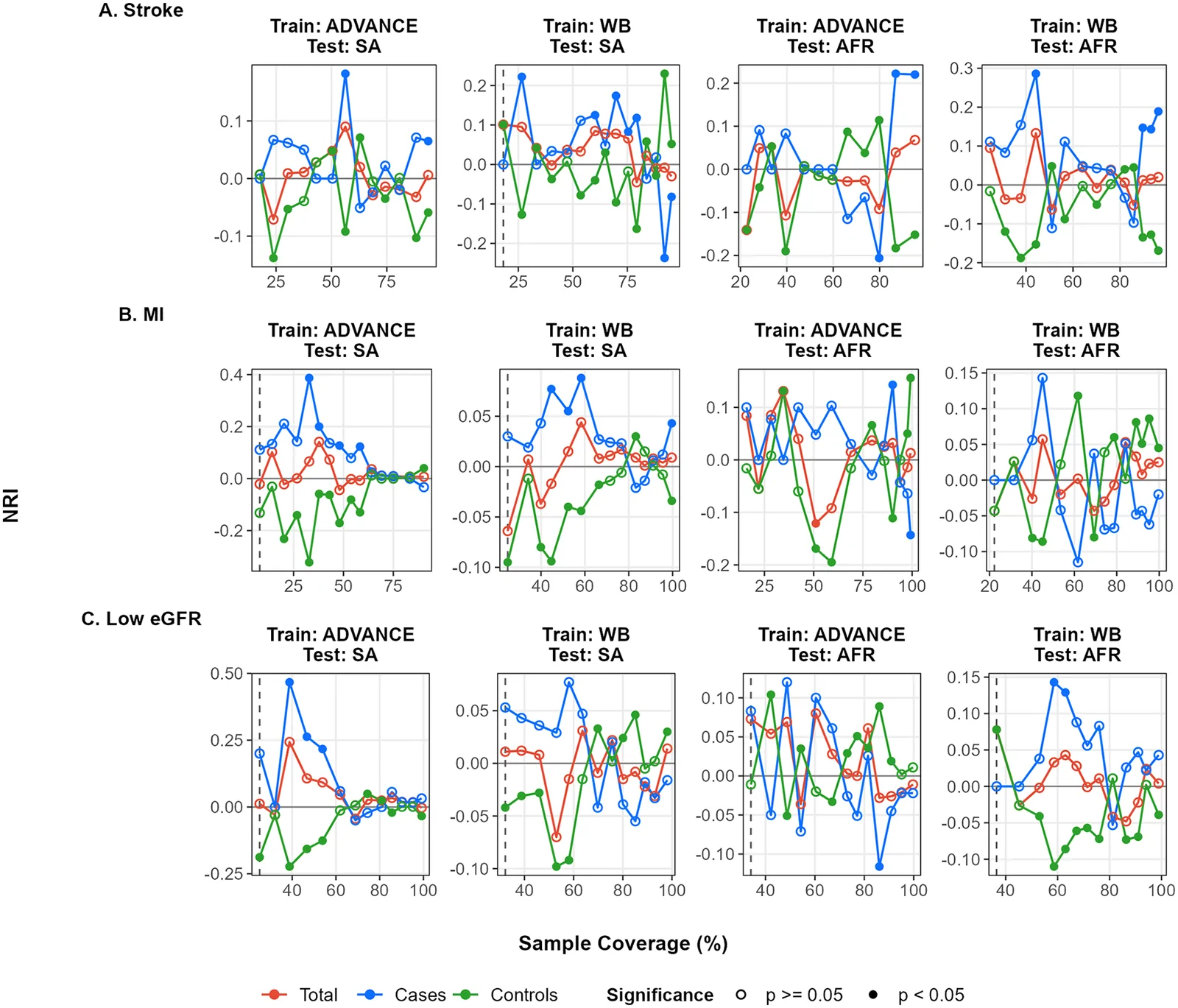
Net reclassification improvement (NRI) for stroke, MI and Low eGFR in the SA and AFR populations.

**Fig 9:**
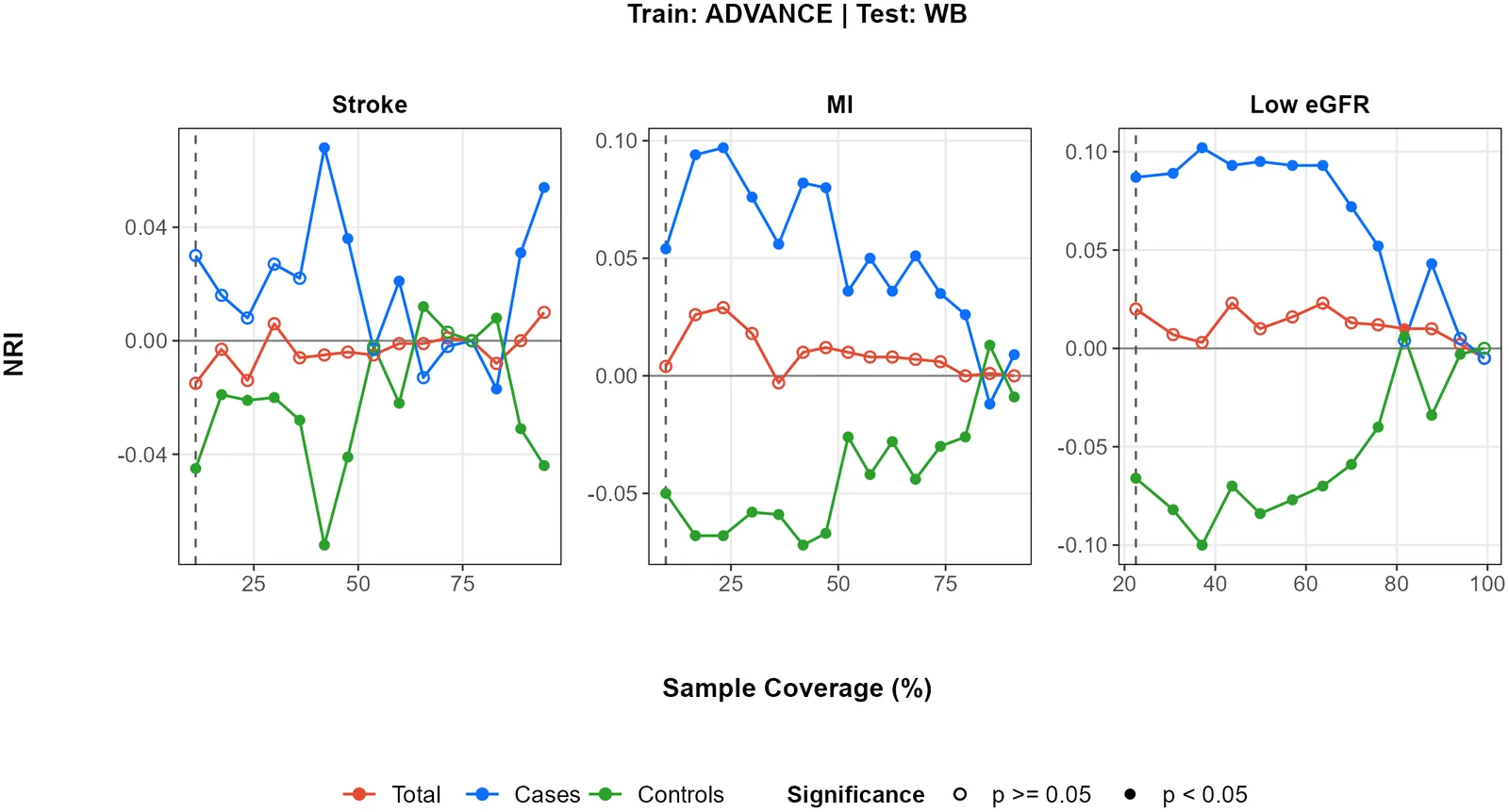
Net reclassification improvement (NRI) in the WB population (ADVANCE-trained). Abbreviations as in Fig 8.

Reclassification performance was evaluated using the NRI. At α ≈ 0.05, total NRI was not significantly different from zero in the majority of evaluable phenotype–population combinations across both training cohorts (Figs 8,9). In the WB population, which had the largest evaluation sample, total NRI ranged from 0.015 to 0.020 (all p ≥ 0.36). A single evaluable combination reached significance (Low eGFR, AFR, WB-trained: total NRI = 0.078, 95% CI: 0.030–0.120, p = 0.004), driven entirely by the non-event component; the event NRI was 0.000 (p = 1.00), with 11 events included. Results for Low eGFR under the race-free definition were consistent (S18 Fig). Complete NRI results across α values, including event and non-event components, are provided in S8 Table.

For each expected error level α, the total NRI and its event (cases) and non-event (controls) components are plotted against sample coverage, comparing MCCP with coverage-matched LR-SP. Filled symbols indicate p < 0.05; open symbols, p ≥ 0.05. The dashed vertical line marks the coverage corresponding to α = 0.05. Panels are arranged by phenotype (rows) and by training cohort and test population (columns). MCCP, Mondrian Cross-Conformal Prediction; LR-SP, logistic regression–based stratification by predicted probabilities; NRI, net reclassification improvement; SA, South Asian; AFR, African/Caribbean.

## Discussion

In this study, we show that integrating Mondrian Cross-Conformal Prediction (MCCP) with polygenic risk scores improves the reliability of genetic risk prediction across ancestries in individuals with type 2 diabetes. By providing calibrated prediction sets with prespecified nominal error control, MCCP enables uncertainty-aware risk stratification while identifying individuals for whom predictions can be made with greater confidence. This property is particularly important when prediction models are applied to heterogeneous populations and imbalanced outcomes [17–19]. To evaluate whether this framework improves the reliability of PRS-based prediction across ancestries, we assessed our previously developed multiPRS under two complementary training settings. In the first, the model was trained in the ADVANCE clinical cohort and evaluated in the WB, SA, and AFR populations, reflecting the typical challenge of transferring PRS-based prediction across cohorts and ancestries. In the second, logistic regression was trained directly in the WB population and evaluated in the SA and AFR populations from the same cohort, allowing us to minimize differences related to study design, recruitment, and healthcare environment, and to test whether the differences observed between populations reflected ancestry and sample size rather than cohort effects.

Across both training frameworks, MCCP and LR-SP achieved comparable discrimination. As expected, performance in the ADVANCE-trained analyses was highest in the WB population, likely reflecting its closer genetic similarity to the European ancestry population used for model development. This observation is consistent with previous studies showing that the transferability of polygenic risk scores generally decreases with increasing genetic distance between populations [26,27]. This equivalence was not limited to the classical cross-cohort setting: when logistic regression was trained directly in the WB population and subsequently evaluated in the SA and AFR populations from the same cohort, the two methods remained comparable across all three phenotypes. Furthermore, stratified downsampling of the WB and SA populations to match the AFR sample size did not materially alter this equivalence. Together, these complementary analyses indicate that the differences observed between populations cannot be explained by the choice of method and instead reflect genetic distance and sample size. The distinct contribution of MCCP therefore lies not in discrimination but in its explicit, prespecified control of the error rate and its identification of individuals for whom no reliable prediction can be made.

Our finding of comparable discrimination between MCCP and LR-SP may appear to contrast with the original MCCP study, which reported that MCCP outperformed empirical top-versus-bottom stratification of the PRS [19]. This difference is explained by the comparator. In the original implementation, individuals were stratified using the raw polygenic score alone, whereas MCCP derived its prediction sets from a model that additionally incorporated age, sex, and genetic principal components. The two rules therefore selected high-confidence individuals from different information, and the advantage attributed to MCCP largely reflected this difference in predictors rather than the conformal step itself.

In our analysis, we removed this asymmetry. Both the MCCP conformal p-values (p0, p1) and the LR-SP probabilities were derived from the same penalized logistic model, trained on the same predictors (the ten weighted PRS, age, sex, diabetes duration, and genetic principal components); they therefore encode the same underlying signal and differ only in how that signal is transformed into a selection rule. As a result, the two rules select largely overlapping subsets, and each selected subset is evaluated identically. Under this matched comparison, discrimination was equivalent, indicating that discriminative performance is governed by the underlying risk model and its predictors rather than by the conformal selection step itself.

This does not diminish the value of MCCP, whose contribution lies in calibrated, prespecified error control and the explicit identification of uncertain predictions that probability-based stratification does not provide.

Notably, the original MCCP study was restricted to European-ancestry samples and identified trans-ethnic application, defined as training in a European population and testing in African or Asian populations, as an important next step [19]. Our work directly addresses this gap by evaluating MCCP when models developed in European-ancestry cohorts are transferred to South Asian and African/Caribbean populations.

The downsampling analysis should nevertheless be interpreted as a sensitivity analysis rather than as evidence that smaller samples intrinsically improve model performance. At low α values, MCCP evaluates performance within a restricted high-confidence subset, and reducing WB and SA to the AFR sample size reduces the absolute number of retained individuals. This can increase the variability of performance estimates, particularly when the retained individuals are highly separable.

Unlike stroke and MI, the relationship between coverage and predictive performance for Low eGFR followed a distinct pattern across populations, likely reflecting the unique genetic and pathophysiological architecture of kidney function and its complex relationship with cardiovascular disease [28]. The method used to estimate kidney function may further contribute to this heterogeneity. Although removing the race coefficient from the CKD-EPI equation shifted the case definition in the AFR population, discrimination remained comparable between methods under both definitions, supporting the robustness of the comparison to evolving clinical classifications of kidney disease. More broadly, ancestry-specific genetic determinants of kidney function [29,30] and the proposed transition toward race-free equations highlight that kidney function should not be treated as a biologically uniform trait across populations, with implications for the generalizability of PRS-based prediction in this domain.

Although AUROC remains one of the most widely used measures of predictive performance, its clinical usefulness may be limited in low-prevalence settings, where high discrimination can coexist with modest positive predictive values. Consistent with this, PPV, NPV, and recall varied across phenotypes and populations, reflecting differences in disease prevalence, class imbalance, and underlying risk distributions [31–33]. The modest PPV values observed in several settings were expected given the low prevalence of these outcomes. Rather than optimizing a single performance metric, MCCP enables prediction to be tailored to a predefined error level (α), making the framework particularly attractive for clinical applications in which the consequences of false-positive and false-negative predictions differ.

The interpretation of MCCP performance also depends on the selected error level. At low α values, MCCP is more selective and restricts predictions to individuals receiving high-confidence singleton predictions, which is the most relevant setting for clinical decision-making. In contrast, when α increases and coverage approaches the full sample, MCCP becomes less selective and includes individuals with lower prediction confidence. Under these high-coverage conditions, performance estimates are expected to converge toward those of LR-SP, and the clinical relevance of the comparison is reduced because the tolerated error rate is substantially higher. Thus, high-coverage results should be interpreted primarily as illustrating the trade-off between coverage and prediction confidence, rather than as the preferred operating point for clinical use.

Beyond discrimination, MCCP also demonstrated generally good calibration, although calibration performance differed across phenotypes. For stroke, empirical miscoverage remained consistently below the nominal error level across both training frameworks, indicating conservative coverage. In contrast, under-coverage was observed for MI in the AFR population and for Low eGFR, where empirical miscoverage exceeded the target error rate. These findings indicate that calibration performance was not uniform across phenotype–population combinations. Clinically, under-coverage implies that the observed prediction error may exceed the user-specified error level for these phenotype–population combinations, reducing confidence in the nominal validity guarantee. This observation is particularly important for underrepresented populations, where reliable uncertainty estimation is essential for equitable clinical implementation. In comparison, LR-SP showed relatively stable Brier scores across phenotypes and training frameworks, including both CKD-EPI equations. However, unlike MCCP, the Brier score summarizes probabilistic accuracy but does not provide formal guarantees that empirical prediction error will remain close to the prespecified α level.

The validity of conformal prediction generally relies on exchangeability between calibration and test observations [34]. In our cross-population analyses, calibration and test observations came from different ancestry groups and, for the ADVANCE-trained models, from different parent cohorts. Ancestry-related differences in linkage disequilibrium, allele frequencies, and genetic architecture can reduce the transferability and accuracy of polygenic scores, which are generally less predictive in non-European populations [8,35]. Differences in risk-factor distributions, event prevalence, and predictor–outcome relationships may further contribute to distribution shift. Under such shift, control of the error rate at the expected level α cannot be assumed a priori and must be assessed empirically in each target population [34].

Our results illustrate this limitation. As described above, empirical error control was generally maintained for stroke but not uniformly for MI or Low eGFR. These findings are compatible with the presence of distribution shift that is not automatically accommodated by standard conformal prediction.

Several features of our design may reduce, without eliminating, the effects of this shift. The predictive models included the first four genetic principal components, which may account for part of the ancestry-related variation in the predictors but do not restore exchangeability between calibration and test populations. The Mondrian construction calibrated cases and controls separately, thereby accommodating class imbalance and targeting class-conditional error control [17], without directly correcting for distribution shift between populations. In the WB-trained analyses, the training, calibration, and test samples came from the same parent cohort, reducing some recruitment- and measurement-related differences, while ancestry-related differences remained. Overall, these findings indicate that conformal error control should be assessed empirically in each target population rather than assumed to transfer automatically from one ancestry group to another.

Beyond calibration, the reclassification analyses were consistent with the equivalence observed in discrimination. Total NRI did not differ significantly from zero in the majority of phenotype–population combinations, including in the WB population, where statistical power was greatest. This indicates that MCCP and coverage-matched LR-SP produce similar binary risk classifications, as expected when both apply a selection rule to the same underlying model. The single combination reaching nominal significance (Low eGFR, AFR, WB-trained) was driven entirely by the non-event component, with no significant effect among events, and should be interpreted with caution given the small number of events. Notably, although the total NRI was close to zero, its components tended to move in opposite directions: MCCP reclassified more cases upward, favouring sensitivity, while probability-based stratification reclassified more controls as low-risk, favouring specificity. These offsetting effects, which cancel in the total, reflect the expected trade-off between sensitivity and specificity when predictions are restricted to a high-confidence subset.

Taken together, these findings indicate that the principal contribution of MCCP lies not in improving discrimination, which was equivalent to LR-SP, but in the guarantees its framework provides. Across two independent training frameworks, MCCP delivered prediction sets with explicit, prespecified control of the error rate, maintained valid calibration in most settings, and identified individuals for whom no reliable prediction could be made. Rather than increasing predictive accuracy, MCCP provides clinicians with explicit information regarding the confidence associated with each prediction, an important property for the future clinical implementation of polygenic risk scores in diverse populations.

While these findings demonstrate the potential of MCCP for improving the reliability of PRS-based prediction across ancestries, several limitations should be considered.

First, MCCP is computationally more demanding than standard logistic regression because it relies on repeated training–calibration cycles. Although runtimes were consistently longer than LR-SP across all phenotype–population combinations, absolute computational requirements remained modest, with memory usage below 200 MB and execution times compatible with routine research applications (S9 Table).

Second, MCCP produces “uncertain” and “unpredictable” predictions that are not directly actionable. The frequency of these prediction sets depends on the selected error level (α), disease prevalence, and the underlying distribution of prediction uncertainty. Consequently, future clinical implementation will require explicit decision pathways defining how these individuals should be managed rather than considering these outputs as prediction failures.

Third, despite the downsampling analyses demonstrating that the observed differences were not explained by sample size alone, the SA and AFR cohorts remained substantially smaller than the WB cohort. Limited numbers of outcome events reduced statistical precision for some phenotype–population combinations and prevented more detailed analyses of ancestry substructure. Continued efforts to recruit participants from underrepresented populations remain essential, particularly as genomic datasets continue to be dominated by individuals of European ancestry [36,37].

Fourth, substantial genetic diversity within African populations limits the generalizability of our findings. African ancestry groups differ considerably in genetic architecture, allele frequencies, admixture patterns, and environmental exposures. PRSs developed in African American populations, who represent only a subset of West African ancestry with admixture, do not necessarily transfer to continental African populations. For instance, genetic and environmental differences between Ugandan (East Africa) and South African cohorts have been shown to further limit the transferability of genetic risk scores derived from African American samples, highlighting the need to adapt PRS to the specific genetic and environmental context of each population [7]. Because our AFR cohort represents a specific UK Biobank African/Caribbean-derived population and sample sizes were insufficient to perform finer ancestry stratification, our conclusions should not be generalized to all African-ancestry populations without additional validation.

Finally, we were unable to explicitly model gene–environment interactions because harmonized information on socioeconomic status, lifestyle, and treatment-related factors was unavailable. Although our control analyses using WB-trained models reduced potential confounding arising from differences between cohorts, environmental and socioeconomic factors may still contribute to differences in prediction performance across ancestry groups within the same country. Future studies incorporating standardized environmental exposures together with genomic information will be important to further improve the transportability, calibration, and clinical applicability of PRS-based prediction models.

Future studies should prospectively validate MCCP-guided risk prediction in independent multi-ancestry cohorts and evaluate its impact on real-world clinical decision-making. Particular attention should be given to larger African, African American, Hispanic/Latino and Asian populations, to improving calibration in phenotype–population combinations showing under-coverage, to comparing MCCP with other uncertainty-aware prediction frameworks, and to developing practical clinical protocols for the management of uncertain and unpredictable predictions.

In conclusion, this study demonstrates that integrating Mondrian Cross-Conformal Prediction with polygenic risk scores improves the reliability of genetic risk prediction across ancestries by explicitly quantifying prediction uncertainty and providing formal control of prediction error in most phenotype–population combinations. Across two complementary training frameworks, MCCP achieved discrimination comparable to logistic regression–based stratification, while adding capabilities that probability-based stratification does not provide: a prespecified, verifiable error level, valid calibration in most phenotype–population combinations, and the explicit identification of individuals for whom no reliable prediction can be made. To our knowledge, this is the first study to evaluate MCCP for cross-ancestry polygenic risk prediction across multiple cardiovascular and renal complications in individuals with type 2 diabetes. Overall, these findings support uncertainty-aware prediction as a promising strategy for improving the equitable application of polygenic risk scores in diverse populations, while emphasizing the importance of continued validation, calibration, and prospective clinical evaluation before widespread implementation.

## Methods

### Ethic statement

The ADVANCE study was approved by the ethics committees of the coordinating centres and each participating center, and only participants who provided written informed consent to the clinical and genetic sub-studies were included in the analysis. Access to UK Biobank data was granted under projects 49731 and 59642, and the present data analysis was approved by the ethics committee of the Centre hospitalier de l’Université de Montréal (CHUM) under the number 2023–11126,22.205-LM. All procedures were conducted in accordance with the Declaration of Helsinki.

In line with our study hypothesis, all analyses were designed to evaluate the predictive performance of the multiPRS across phenotypes (stroke, MI, Low eGFR (< 60 ml/min/1.73m²)) and ancestral groups (WB, SA, and AFR). Performance metrics (AUROC, PPV, NPV, recall, sample coverage) were systematically reported by phenotype and ancestry. For the Mondrian Cross-Conformal Prediction (MCCP) analyses, the main results were presented at an error level of α = 0.05, corresponding to a 95% confidence threshold, while complementary figures illustrate the variation of performance metrics across the full α range (0–1) for visualization purposes.

Our multiPRS predictive model was based on 598 SNPs identified by summary statistics of meta-analyses of publicly available genome-wide association studies (GWAS) gathering genomic and phenotypic information from over one million individuals of European descent [13]. We combined 10 weighted polygenic risk scores (PRS) gathering genomic variants associated to cardiovascular and renal diseases alongside their key risk factors, the first four principal component of ethnicity, sex, age at onset and diabetes duration into one logistic regression (LR) model, to predict renal and cardiovascular complications of T2D in a single prediction model using samples and data from European participants of T2D of the ADVANCE trial [13,38]. Genotyping and imputation of the ADVANCE samples were performed as described in [6]. Details on the construction and the multi-PRS model and list of SNPs are available in the Supplementary Material of our previous study [6].

### Origin and selection of SNPs

In accordance with our previous study (Diabetologia 2021) and its complementary material (ESM), we selected 598 SNPs from 47 GWAS/meta-analyses according to a step-by-step procedure: (i) systematic identification of GWAS (NHGRI-EBI, HuGE, literature); (ii) definition of bond imbalance blocks (R² ≥ 0.8; R² ≥ 0.7 for proxies if necessary); (iii) selection of the main SNP by locus/trait and harmonization of effects. Some SNPs may appear in more than one wPRS when they have been associated with distinct traits. The exact SNP identifiers are listed in the Supplementary Materials of reference [6], which also describes the full construction methodology; the individual effect sizes, however, are not publicly available.

### Cohorts

**ADVANCE** (Clinical trial number: NCT00145925, registered on ClinicalTrials.gov, on September 2, 2005) was a 2x2 factorial design, randomized controlled trial of blood pressure (BP) lowering (perindopril-indapamide *vs* placebo) and intensive glucose control (gliclazide MR-based intensive intervention with a target of 6.5 HbA1c *vs* standard care) in patients with T2D. A total of 11,140 participants were recruited from 215 centers in 20 countries from Asia, Australia, North America and across Europe. They were older than 55 years and diagnosed with T2D after the age of 30 years. We used a subset of 4,098 genotyped T2D patients of genetically determined European descent followed for a period of 4.5 years in ADVANCE [13].

**UK Biobank**: This study was performed under projects 49731 and 59642. The UK Biobank study started in 2006 and, until 2010, recruited more than 500,000 participants from the general UK population, aged between 40–69 [39]. Individuals had type 2 diabetes diagnosed by a doctor (Field ID 2443) at the age of 30 years or older to avoid potential type1 diabetes. We included 17,574 individuals with T2D who self-identified as White British (WB), and 1,145 South-Asian (SA) and 749 African (AFR) participants whose genetic ancestry was confirmed by UK Biobank genetic principal components (Field ID 22006).

Phenotypic data collected included year of birth (Field ID 34), age at recruitment (Field ID 21022), sex (Field ID 31), genetic sex (Field ID 22001), age diabetes diagnosed (Field ID 2976), age when attended assessment centre (Field ID 21003). These last 2 phenotypes allowed us to calculate the duration of diabetes (derived phenotype). eGFR, calculated by the CKD-Epi formula, used plasma creatinine levels (Field ID 30510). Albuminuria (Field ID 30500) and creatininuria (Field ID 30700) were used to calculate micro and macroalbuminuria. For systolic blood pressure, we used the automated reading (Field ID 4080), same for diastolic blood pressure (Field ID 4079), medications blood pressure or diabetes or exogenous hormones (Field ID 6153), and medications for cholesterol, blood pressure or diabetes (Field ID 6177) were used to define hypertensive participants. The samples from the UKBB were genotyped and imputed as described in our publication [6]. Around 35,000 SNPs have been used to calculate principal components (PCs) for each patient as previously reported [6]. PCs vectors were used as covariates.

In ADVANCE, the genotyped subset (N = 4,098) included European genetic ancestry only and lacked ancestry categories comparable to UK Biobank; consequently, ancestry-stratified evaluations were performed in UK Biobank only (WB, SA, AFR), while ADVANCE was used for model development and calibration.

The clinical outcomes (stroke and MI) were based on physician-confirmed diagnoses recorded at baseline and during an average follow-up of 4–6 years in both the ADVANCE and UK Biobank cohorts.

### Statistical and machine learning-based prediction models

Various statistical methods, both traditional logistic regression (LR) models and modern machine learning algorithms, were used to evaluate the predictive performance of the multiPRS for each outcome. For this purpose, we used the PyCaret program, an open-source machine learning library in Python [40]. The cohort used to train the models was ADVANCE, while each of the UK Biobank populations (WB, SA, and AFR) was used separately for testing. A 5-fold cross-validation procedure was applied during model training and calibration. The input features included in the analysis were the 10 weighted PRS (wPRS), the age of T2D diagnosis, diabetes duration, patient sex, as well as the first four principal components (PC 1–4) of genetic ancestry. The statistical and machine learning methods tested are: CatBoost Classifier, Extreme Gradient Boosting (XGBoost), Decision Tree Classifier, Dummy Classifier, Extra Trees Classifier, Gradient Boosting Classifier, K Neighbors Classifier, Light Gradient Boosting Machine, Linear Discriminant Analysis, Logistic Regression, Naive Bayes, Quadratic Discriminant Analysis, Random Forest Classifier, Ridge Classifier, SVM - Linear Kernel, Ada Boost Classifier. To evaluate the performance of each model, we calculated and compared the AUROC with the confidence intervals using the PyCaret package.

### MCCP analysis

Conformal prediction (CP) is a method used to estimate the confidence of predictions for new instances, based on historical data sets. Rather than providing a single-point prediction, CP produces a set of possible outcomes with a validity guarantee: under the assumption that data are exchangeable, the true label lies within the prediction region with probability at least 1 − α, where α is a user-defined error level [18,41].

A key component of CP is the Nonconformity Measure (NCM), which quantifies how unusual or nonconforming an observation is with respect to the model built from the training data. The NCM is calculated for each individual in both the calibration and test sets and serves as the basis for determining which labels should be included in the prediction region [18]. CP is model-agnostic and can be applied on top of any machine learning algorithm that produces predictive outputs.

In this study, we implemented MCCP using LR as the base model, following the CP framework.

We used the ADVANCE European ancestry population as a training population to build and calibrate the model, while the UKBB WB, AFR and SA populations were used separately as test populations to estimate probabilities and evaluate model performance. The training set was randomly divided into k folds (k=5). For each fold, k−1 subsets were used as the training set to fit an LR model using R’s glmnet package, while the remaining subset was used as the calibration set. A prediction region was defined for each individual using the MCCP method, which accounts for class imbalance [17,19]. Based on the NCM, a prediction region was computed for each test observation.

The Non-Compliance Measure  NCMy=−y·d(x), where d(x) is the decision value of the fitted model (here, logistic regression). On the calibration set, for an individual i and a class y (case/control), the p-value is [19]:


$$\begin{array}{c} p_{y}^{i}=\frac{\left|\left\{j\in \{\text{1},\ldots ,N_{\text{cal}}\}:\text{NC}{\text{M}}_{\text{y},\text{j}}\geq \text{NC}{\text{M}}_{\text{y},\text{i}}\right\}\right|}{N_{\text{cal}}+\text{1}} \end{array}$$

α level region is$\Gamma^{\alpha }=\left\{y\in Y:p_{y}>\alpha \right\}.$

Depending on the class-specific conformal p-values p1 (case) and po (control), and user-defined error level α the prediction can be classified as: case (p1> α and p0≤ α), control (p0 > α and p1 ≤ α), uncertain(p1 > α and p0 > α), or unpredictable (p1 ≤ α and p0 ≤ α) with a confidence level of 1−α, credibility corresponds to the probability associated with the most probable class, i.e., the max (p1, p0)[17,19]. Coverage refers to the proportion of individuals for whom the model issues a confident prediction (i.e., classified as either case or control), excluding uncertain or unpredictable classifications.

### Logistic Regression - based Stratification by Predicted Probabilities (hereafter referred to as LR-SP)

For the LR-SP model, individuals were stratified using the out-of-sample predicted probabilities from a penalized logistic regression (elastic-net, R package glmnet) trained on the same ten polygenic risk scores and covariates (sex, age at diagnosis, diabetes duration, and PC1–PC4) as the MCCP base model. Rather than stratifying on the raw polygenic score, individuals whose predicted probabilities fell in the upper and lower quantiles were classified as high- and low-risk, respectively. The two tails were defined symmetrically so that the resulting coverage matched that of MCCP at each operating point, enabling a comparison in which both methods drew on the same predictive information and differed only in their selection mechanism.

### Evaluation of discriminative performance

Following the evaluation approach of Sun et al. [19], discriminative performance within each selected subset was assessed by refitting a logistic model including the same ten polygenic risk scores and covariates (sex, age at diagnosis, diabetes duration, and PC1–PC4), and computing the AUROC, PPV, NPV, and recall from its predictions. The same refitting procedure was applied identically to the subsets selected by MCCP and by LR-SP, so that any difference in performance reflected the selection mechanism rather than the evaluation. These metrics were computed only at operating points where the selected subset contained at least 10 cases and 10 controls, applied identically across all phenotypes and populations; because prevalence varied across phenotypes, this criterion corresponded to different subset sizes, and in smaller cohorts the lowest evaluable error level exceeded α = 0.05. Confidence intervals for AUROC, PPV, NPV, and recall were obtained using the pROC package in R.

## Calibration assessment

### Expected versus observed error

Calibration was evaluated by comparing the observed error with the expected error level, α. For each value of αfrom 0 to 1, in increments of 0.01, the observed error was calculated over the full test sample [19].

For MCCP, an error occurred when the conformal p-value associated with the true class was less than or equal to α:


$$\begin{array}{c} {\text{Observed}}_{\text{err}}(\text{MCCP};\alpha )=\frac{\text{1}}{n}\sum_{i=\text{1}}^{n}I\left(p_{y_{i}}^{i}\leq \alpha \right). \end{array}$$

For LR-SP, the analysis used the out-of-sample predicted probabilities from the underlying logistic regression model, before applying the upper- and lower-tail selection used in the performance analyses. An error occurred when a control had a predicted probability greater than 1−αor a case had a predicted probability lower than α:


$$\begin{array}{c} {\text{Observed}}_{\text{err}}(\text{LR}-\text{SP};\alpha )=\frac{\text{1}}{n}\sum_{i=\text{1}}^{n}I\left[\left(p^{i}>\text{1}-\alpha \text{ and }y_{i}=\text{0}\right)\text{ or }\left(p^{i}<\alpha \text{ and }y_{i}=\text{1}\right)\right]. \end{array}$$

### Empirical miscoverage (MCCP)

For MCCP, calibration was quantified as conformal validity. The empirical miscoverage at error level α was defined as the proportion of individuals whose true label fell outside the conformal prediction region. Empirical miscoverage at error level α was defined as


$$\begin{array}{c} \text{Miscoverage}(\alpha )=\frac{\text{1}}{n}\sum_{i}\text{1}\left[(Y_{i}=\text{1}\wedge p_{\text{1}i}\leq \alpha )\text{ or }(Y_{i}=\text{0}\wedge p_{\text{0}i}\leq \alpha )\right] \end{array}$$

where $p_{\text{0}i}$and $p_{\text{1}i}$denote the conformal p-values for the control and case classes, respectively, and 1[·]is the indicator function.

The validity gap was calculated as


$$\begin{array}{c} \text{Validity Gap}(\alpha )=\text{Miscoverage}(\alpha )-\alpha . \end{array}$$

For logistic regression–based stratification (LR-SP), probabilistic calibration was assessed using the Brier score, computed on the subset of individuals selected from the upper and lower tails of the predicted probability distribution, with coverage matched to that of MCCP at the corresponding α level:


$$\begin{array}{c} \text{Brier}(\alpha )=\frac{\text{1}}{m}\sum_{i\in S}(Y_{i}-q_{i}{)}^{\text{2}}, \end{array}$$

where Sdenotes the subset selected by LR-SP at matched coverage, mis the subset size, and $q_{i}$ is the predicted probability from the logistic regression model.

Because conformal validity (miscoverage) and probabilistic calibration (Brier score) quantify different properties, calibration was assessed for each method using the metric most appropriate for its prediction framework, and the two measures are therefore not directly comparable.

### Net Reclassification Improvement (NRI)

Reclassification between MCCP and LR-SP was compared using the Net Reclassification Improvement (NRI), with 95% confidence intervals estimated using the bootstrap percentile method (500 bootstrap resamples) [42]. At a given expected error level α, MCCP first identifies individuals receiving a confident singleton prediction, defined by (p1 > α and p0 ≤ α) or (p0 > α and p1 ≤ α); individuals assigned to the uncertain or empty regions were excluded. Binary risk classifications were then derived for both methods using the same procedure: within each selected subset, a logistic model was refitted and its predicted probabilities were thresholded at the empirically optimal (Youden) cut-off, applied identically to MCCP and to the coverage-matched LR-SP subset. The conformal p-values were used only to define the confident subset, not to compute the reclassification. Subsets with fewer than 10 cases or 10 controls were not evaluated, and points at which the refitted model perfectly separated the data were excluded.

The overall NRI was defined as


$$\begin{array}{c} NRI=[Pr(↑|Y=1)-Pr(↓|Y=1)]+[Pr(↓|Y=0)-Pr(↑|Y=0)], \end{array}$$

where ↑ denotes individuals classified as cases by MCCP and as controls by LR-SP, whereas ↓ denotes individuals classified as controls by MCCP and as cases by LR-SP.

### Code availability

All scripts used in this study, including the MCCP implementation, the LR-SP analyses, the downsampling procedure, the statistical analyses, and the figure-generation scripts are openly available in a public GitHub repository (https://github.com/edoh-kodji/mccp-lrsp-multiprs-ancestry) and archived on Zenodo with a persistent DOI (https://doi.org/10.5281/zenodo.21416613).

## Supporting information

S1 Fig AUROC of 16 machine learning algorithms for stroke, MI, and Low eGFR prediction. Models were trained on the ADVANCE cohort and evaluated across UK Biobank populations. MI, myocardial infarction; Low eGFR, estimated glomerular filtration rate based on the CKD-EPI 2009 formula (<60 ml/min/1.73m²); AUROC, area under the receiver operating characteristic curve; AFR, African/Caribbean; SA, South Asian; WB, White British.
(TIF)

S2 Fig PPV, NPV, and recall of MCCP versus LR-SP in the ADVANCE-trained model tested in WB. Rows show the performance metric (PPV, NPV, and recall), and columns show the phenotype (stroke, MI, and Low eGFR). See Fig 1 for axis details.
(TIF)

S3 Fig PPV of MCCP versus LR-SP across SA and AFR populations. See Fig 1 for axis details. Low eGFR was defined using the CKD-EPI 2009 equation including the race coefficient.
(TIF)

S4 Fig NPV of MCCP versus LR-SP across SA and AFR populations. See Fig 1 for axis details. Low eGFR was defined using the CKD-EPI 2009 equation including the race coefficient.
(TIF)

S5 Fig Recall of MCCP versus LR-SP across SA and AFR populations. See Fig 1 for axis details. Low eGFR was defined using the CKD-EPI 2009 equation including the race coefficient.
(TIF)

S6 Fig PPV, NPV, and recall of MCCP versus LR-SP for Low eGFR with and without the race coefficient in AFR. See Fig 1 for axis details.
(TIF)

S7 Fig Covariate distributions before and after downsampling in the WB population for stroke. Density distributions are shown for the original and downsampled datasets. Continuous covariates include age at diabetes diagnosis, diabetes duration, and the first four genetic principal components (PC1–PC4). Downsampling was stratified by case–control status using the AFR sample size as the reference while preserving phenotype-specific class balance.
(TIF)

S8 Fig Covariate distributions before and after downsampling in the WB population for MI. See S7 Fig legend for details.
(TIF)

S9 Fig Covariate distributions before and after downsampling in the WB population for Low eGFR. See S7 Fig legend for details.
(TIF)

S10 Fig Covariate distributions before and after downsampling in the SA population for stroke. See S7 Fig legend for details.
(TIF)

S11 Fig Covariate distributions before and after downsampling in the SA population for MI. See S7 Fig legend for details.
(TIF)

S12 Fig Covariate distributions before and after downsampling in the SA population for Low eGFR. See S7 Fig legend for details.
(TIF)

S13 Fig PPV of MCCP versus LR-SP after downsampling WB and SA to the AFR sample size. Rows show stroke, MI, and Low eGFR, and columns show the test population (WB, SA, and AFR). WB and SA were downsampled by stratified random subsampling to match the AFR sample size, whereas AFR was not downsampled and is shown as the reference population. See Fig 4 for panel layout and Fig 1 for axis details.
(TIF)

S14 Fig NPV of MCCP versus LR-SP after downsampling WB and SA to the AFR sample size. Rows show stroke, MI, and Low eGFR, and columns show the test population (WB, SA, and AFR). WB and SA were downsampled by stratified random subsampling to match the AFR sample size, whereas AFR was not downsampled and is shown as the reference population. See Fig 4 for panel layout and Fig 1 for axis details.
(TIF)

S15 Fig Recall of MCCP versus LR-SP after downsampling WB and SA to the AFR sample size. Rows show stroke, MI, and Low eGFR, and columns show the test population (WB, SA, and AFR). WB and SA were downsampled by stratified random subsampling to match the AFR sample size, whereas AFR was not downsampled and is shown as the reference population. See Fig 4 for panel layout and Fig 1 for axis details.
(TIF)

S16 Fig Expected versus observed error for Low eGFR in the AFR population, with and without the race coefficient. For each expected error level α (0–1), the observed error is plotted against α; the diagonal indicates perfect calibration. Low eGFR was defined using the CKD-EPI 2009 equation including the race coefficient; Low eGFR (AS) was defined using the race-free CKD-EPI 2021 equation. Results are shown under both the ADVANCE-trained and WB-trained models. Abbreviations as in Fig 5.
(TIF)

S17 Fig Empirical miscoverage of MCCP and Brier score of LR-SP for Low eGFR in the AFR population, with and without the race coefficient, at α = 0.05. Measures are shown as in Fig 6. Low eGFR was defined using the CKD-EPI 2009 equation including the race coefficient; Low eGFR (AS) using the race-free CKD-EPI 2021 equation, under both training cohorts. Abbreviations as in Fig 6.
(TIF)

S18 Fig Net reclassification improvement (NRI) for Low eGFR in the AFR population, with and without the race coefficient. Low eGFR was defined using the CKD-EPI 2009 equation including the race coefficient; Low eGFR (AS) using the race-free CKD-EPI 2021 equation. Abbreviations as in Fig 8.
(TIF)

S1 Table Reclassification of Low eGFR status with and without the race coefficient in AFR. Low eGFR was defined using the CKD-EPI 2009 equation including the race coefficient, whereas Low eGFR (AS) was defined using the race-free CKD-EPI 2021 equation. Both equations were applied to the same 713 AFR individuals.
(XLSX)

S2 Table Complete performance of MCCP and LR-SP across all evaluable error levels (α) in the ADVANCE-trained model tested in WB. Values are reported for every evaluable operating point across the full α range, rather than only the representative low- and high-coverage points shown in Table 2. For MCCP, coverage corresponds to the proportion of individuals receiving a singleton prediction at the specified expected error level α; for LR-SP, coverage was matched to MCCP by selecting individuals from the top and bottom tails of the predicted risk distribution. Operating points were evaluated only where the selected subset contained at least 10 cases and 10 controls. AUROC, area under the receiver operating characteristic curve; PPV, positive predictive value; NPV, negative predictive value; Recall, sensitivity among cases included in the predicted subset; MCCP, Mondrian Cross-Conformal Prediction; LR-SP, logistic regression–based stratification by predicted probabilities; WB, White British.
(XLSX)

S3 Table Complete performance of MCCP and LR-SP for stroke across all evaluable error levels (α) in the SA and AFR populations. Values are reported for every evaluable operating point across the full α range. See S2 Table legend for column and abbreviation details. SA, South Asian; AFR, African/Caribbean.
(XLSX)

S4 Table Complete performance of MCCP and LR-SP for MI across all evaluable error levels (α) in the SA and AFR populations. Values are reported for every evaluable operating point across the full α range. See S2 Table legend for column and abbreviation details.
(XLSX)

S5 Table Complete performance of MCCP and LR-SP for Low eGFR across all evaluable error levels (α) in the SA and AFR populations, with and without the race coefficient. Values are reported for every evaluable operating point across the full α range. Low eGFR was defined using the CKD-EPI 2009 equation including the race coefficient; Low eGFR (AS) using the race-free CKD-EPI 2021 equation. See S2 Table legend for column and abbreviation details.
(XLSX)

S6 Table Statistical comparison of covariate distributions between the original and downsampled datasets, by phenotype (stroke, MI, Low eGFR) in the WB and SA populations. Continuous covariates were compared using the Kolmogorov–Smirnov (KS) test, and binary variables using Fisher’s exact test. P-values greater than 0.05 indicate no significant difference in distribution, suggesting that the covariate structure was preserved following downsampling.
(XLSX)

S7 Table Complete performance of MCCP and LR-SP across all evaluable error levels (α) after downsampling WB and SA to the AFR sample size. Values are reported for every evaluable operating point across the full α range. WB and SA populations were downsampled via stratified random subsampling to match the AFR sample size, whereas the AFR population was not downsampled and is shown as the reference. See S2 Table legend for column and abbreviation details.
(XLSX)

S8 Table Event, nonevent, and total Net Reclassification Improvement (NRI) comparing MCCP and LR-SP across all training populations, testing populations, phenotypes, and error rate (α). NRI is decomposed into its event and non-event components alongside the total, at each α level. See S2 Table legend for population and abbreviation details.
(XLSX)

S9 Table Computational cost of MCCP and LR-SP across phenotypes, training populations, and testing populations. Runtime is reported as the total execution time (seconds) and normalized per 1,000 test individuals to facilitate comparisons across datasets of different sizes. Peak memory corresponds to the maximum RAM used during model execution (MB, megabytes). MCCP training-calibration cycles correspond to the total number of model fitting procedures performed during cross-conformal prediction (5 folds × 21 elastic-net α values = 105 cycles). All analyses were performed on a single CPU core.
(XLSX)

## Abbreviations

ADVANCE: Action in Diabetes and Vascular Disease; Preterax and Diamicron Modified Release Controlled Evaluation
AFR: African/Caribbean ancestry (UK Biobank)
CP: Conformal Prediction
LR-SP: Logistic Regression–based Stratification by Predicted Probabilities
MCCP: Mondrian Cross-Conformal Prediction
NCM: Nonconformity Measure
MultiPRS: Multi-Polygenic Risk Score
SA: South Asian ancestry (UK Biobank)
WB: White British ancestry (UK Biobank)
wPRS: Weighted Polygenic Risk Score
